# Biodegradation of Polyethylene Terephthalate (PET) microplastics in a Microbial Fuel Cell

**DOI:** 10.1007/s11274-026-05374-2

**Published:** 2026-11-04

**Authors:** Merin Thyagu Pereira, Tajalli Keshavarz, Godfrey Kyazze

**Affiliations:** Sustainable Biotechnology Research Group, School of Life Sciences, University of Westminster, London, UK

**Keywords:** *Ideonella sakaiensis*, *Shewanella oneidensis*, Bioelectrochemical, Microplastics, Biodegradation, Microbial fuel cell

## Abstract

Plastics persist in the environment for centuries, releasing harmful micro- and nanoplastics (MNPs) that threaten ecosystems and may be detrimental to human health. This study investigated the feasibility of a bioelectrochemical approach for the biodegradation and upcycling of polyethylene terephthalate (PET) microplastics. Over 40 days, three microbial fuel cell (MFC) systems were evaluated: (i) *Shewanella oneidensis* monoculture, (ii) *Ideonella sakaiensis* monoculture and (iii) their coculture. The *S. oneidensis* monoculture achieved 9.2% (w/w) PET biodegradation with acetic acid as a metabolite and a power density of 1.01 mW m⁻². The *I. sakaiensis* monoculture achieved 35.2% (w/w) PET biodegradation with acetic acid and ethanol as metabolites with a power density of 1.17 mW m⁻². The coculture exhibited moderate PET biodegradation (16.4% w/w) and produced acetic acid and ethanol, and a lower power density of 0.202 mW m⁻². These findings demonstrate that *Ideonella sakaiensis* can anaerobically ferment PET microplastics as the sole carbon source into metabolites including acetic acid and ethanol, while concurrently exhibiting bioelectrochemical activity. Notably, the electroactive bacterium *S. oneidensis* also exhibited PET-associated biodegradation, supported by PET mass loss and surface deterioration, alongside acetic acid formation and bioelectricity generation, highlighting its potential for the simultaneous biodegradation and bioelectrochemical upcycling of PET microplastics. However, cocultivation of *I. sakaiensis* and *S. oneidensis* reduced both PET biodegradation and power generation relative to the best-performing monoculture, suggesting that interspecies competition, antagonistic interactions or metabolic imbalance may limit synergistic activity. Overall, this study demonstrates the potential of MFCs for the simultaneous biodegradation and bioelectrochemical upcycling of PET microplastics, while highlighting the importance of microbial interactions in determining process efficiency.

**Supplementary Information:**

The online version contains supplementary material available at https://doi.org/10.1007/s11274-026-05374-2.

## Introduction

Polyethylene terephthalate (PET) is one of the most widely used thermoplastic polymers due to its excellent mechanical strength, chemical stability and durability (Bharadwaj *et al.* 2024). However, these same properties confer resistance to biodegradation, allowing discarded PET to persist in the environment and gradually fragment into microplastics that accumulate in terrestrial and aquatic ecosystems (Bergeson *et al*. 2024; Liu *et al*. 2024; Hadji-Thomas *et al*. 2025). The environmental prevalence of MNPs is reflected by their detection and uptake across diverse biological systems, including crop plants (Li *et al*. 2020), soil organisms (Bodor *et al*. 2024) and aquatic species (Besseling *et al*. 2015). Human exposure may occur predominantly through ingestion and inhalation, with microplastics reported in drinking water (Schymanski *et al*. 2018) and subsequently detected in human tissues, including the placenta (Ragusa *et al*. 2021), liver and brain (Nihart *et al*. 2025). Their presence in human tissues and the potential of smaller particles to translocate across biological barriers have raised concerns regarding possible systemic and neurological health effects (Hadji-Thomas *et al*. 2025). Exposure to MNPs has been associated with oxidative stress, inflammation, cellular dysfunction and immune dysregulation (Thapliyal *et al*. 2025), while plastic-associated additives and adsorbed environmental contaminants may contribute additional chemical toxicity (Hong and Kim 2024).

Despite these environmental and health concerns, PET waste represents an abundant carbon resource with considerable potential for biological valorisation into value-added chemicals, materials and bioenergy, thereby contributing to a circular bioeconomy (Pilapitiya and Ratnayake 2024; Han *et al*. 2025; Yang *et al*. 2026). Advances in microbial and enzymatic PET depolymerisation have therefore highlighted biological recycling as a promising sustainable alternative to conventional recycling technologies (Amalia *et al*. 2023; Liu *et al*. 2025). Microbial plastic degradation can potentially combine environmental remediation with the conversion of polymer-derived carbon into useful products; however, polymer degradation remains a major rate-limiting step in integrated microbial degradation and upcycling processes (Weldon *et al*. 2025). Recent advance in microbial plastic biotechnology have therefore increasingly focused on coupling polymer depolymerisation with microbial conversion of the resulting monomers and intermediates into value-added products (Pérez-García *et al*. 2025). Nevertheless, broader application remains constrained by factors including PET crystallinity, limited accessibility of the polymer chains and the catalytic efficiency of naturally occurring PET-degrading enzymes (Liu *et al*. 2025).

Microbial Fuel Cells (MFCs) provide a bioelectrochemical platform for coupling microbial metabolism with energy recovery. In an MFC, microorganisms oxidise available organic substrates and transfer metabolically derived electrons to an anode through extracellular electron transfer (EET) process, generating electrical current through the external circuit. *Shewanella oneidensis *MR-1 was selected as the electroactive partner because of its facultative anaerobic metabolism and well-characterised extracellular electron-transfer (EET) pathways, which enable metabolically derived electrons to be transferred to extracellular electron acceptors, including electrodes (Zang *et al*. 2023; Ford and TerAvest 2023). Extending this concept to plastic waste is particularly attractive because microbial or enzymatic depolymerisation can potentially transform recalcitrant polymers into soluble intermediates that become available for microbial metabolism and subsequent bioelectrochemical conversion. Thus, integrating PET-degrading microorganisms with electroactive bacteria offers a potential strategy for linking polymer degradation with recovery of PET-derived chemical energy as bioelectricity.

*Ideonella sakaiensis *201-F6^T^ is a well-characterised PET-degrading bacterium that depolymerises PET through the sequential action of PETase and MHETase (Yoshida *et al*. 2016). Under aerobic conditions, degradation remains relatively slow, with a degradation rate of approximately 0.13 mg cm⁻² day⁻¹ reported for low-crystallinity (1.9%) PET film at 30 °C (Yoshida *et al*. 2016). Importantly, *I. sakaiensis* is a facultative anaerobe capable of degrading PET under anaerobic conditions and fermenting PET-derived carbon into metabolites including acetate and ethanol (Kalathil *et al*. 2022). This metabolic capability provides the rationale for integrating PET biodegradation with MFCs, in which soluble metabolites generated during PET fermentation could potentially serve as electron donors for subsequent bioelectrochemical processes.

Kalathil *et al*. (2022) provided proof-of-concept for this approach by demonstrating anaerobic PET fermentation by *I. sakaiensis* followed by bioelectricity generation by coculturing it with the established exoelectrogenic bacterium *Geobacter sulfurreducens*. In this system, *I. sakaiensis* fermented PET-derived carbon to acetate and ethanol, while acetate was subsequently utilised by *G. sulfurreducens* for bioelectricity generation. Importantly, *I. sakaiensis* primarily functioned as a PET-degrading and fermentative organism, converting PET-derived carbon into metabolites that could support subsequent bioelectrochemical processes when coupled with an electroactive partner (Kalathil *et al*. 2022). Consequently, the direct contribution of *I. sakaiensis* to electrochemical activity and EET in MFCs remains largely unexplored.

Building on this concept, *Shewanella oneidensis *MR-1 was selected in the present study as an alternative electroactive partner for *I. sakaiensis. S. oneidensis* is a facultative anaerobe with well-characterised EET pathways that enable metabolically derived electrons to be transferred to extracellular electron acceptors, including electrodes (Fapetu *et al*. 2016; Zang *et al*. 2023; Ford and TerAvest 2023). The coculture was therefore designed to investigate whether complementary microbial functions - PET depolymerisation and fermentation by *I. sakaiensis* and EET by *S. oneidensis* - could couple PET microplastic biodegradation with bioelectricity generation. Separate monoculture MFCs were included to determine the individual contribution of each organism and thereby provide a basis for interpreting their interaction and performance in coculture. The *I. sakaiensis* monoculture enabled PET degradation, fermentation and potential electrochemical activity to be assessed independently of an established electroactive partner, whereas the *S. oneidensis* monoculture enabled its electrochemical activity and potential contribution to PET-associated degradation to be evaluated separately. Although *S. oneidensis* extensively characterised as an electroactive microorganism, direct PET-degrading activity by wild type *S. oneidensis* MR-1 remains largely unexplored.

Accordingly, this study investigated the feasibility of simultaneous PET microplastic biodegradation and bioelectricity generation in MFCs containing *I. sakaiensis*, *S. oneidensis*, or their coculture. Unlike conventional MFC studies employing readily metabolised co-substrates, no additional carbon source was supplied, minimising confounding effects and enabling the relationship between PET degradation, microbial metabolism and bioelectricity generation to be investigated. Specifically, the study aimed to determine whether (i) *I. sakaiensis* could couple anaerobic PET biodegradation and fermentation with measurable electrochemical activity, (ii) *S. oneidensis* could contribute to PET-associated biodegradation while generating bioelectricity, and (iii) cocultivation of the two organisms could enhance PET degradation and energy recovery through complementary metabolic activities. This approach therefore evaluates the potential of MFCs as an integrated platform for the bioremediation and bioelectrochemical upcycling of PET microplastics.

## Materials and methods

### Microorganisms and culture conditions

**Fig. 1:**
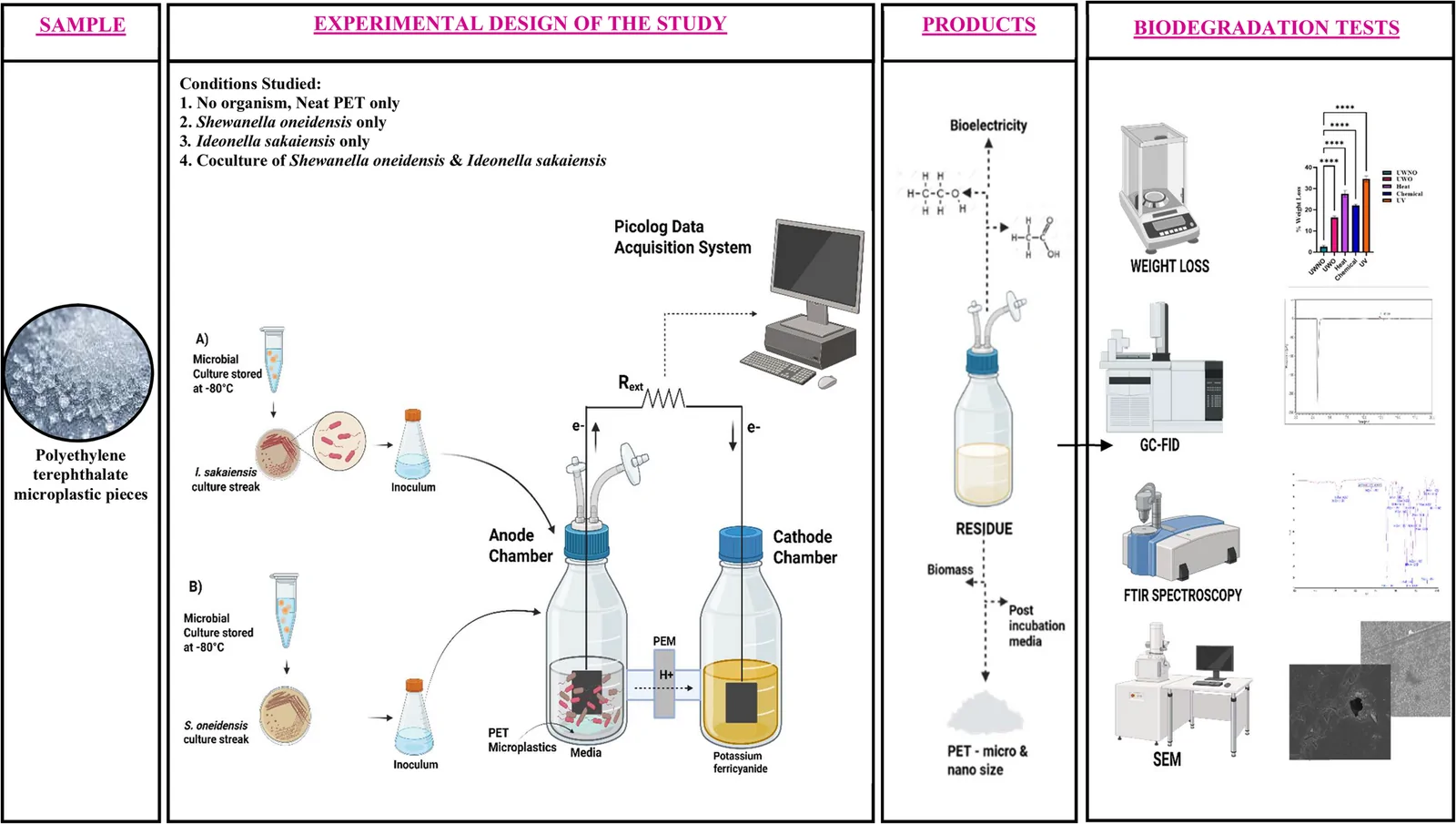
Schematic illustration of the experimental design used in the study

Cryopreserved *Ideonella sakaiensis* 201-F6^T^ (NBRC 110686​) and *Shewanella oneidensis* MR-1 (NCIMB 14063) from the University of Westminster’s culture collection were revived in NBRC 802 and LB media, respectively, and incubated overnight at 30 °C, 180 rpm for mono- and co-cultures. The exponential-phase cultures of *Shewanella oneidensis* MR-1 and *Ideonella sakaiensis* 201-F6^T^, characterised by OD 0.2 at 600 nm, were used for the studies. Ultrapure water (18.2 MΩ· cm at 25 °C) was used for all experiments unless otherwise stated (Fig. 1).

### Preparation of anolyte and catholyte

Bicarbonate-buffered medium (BBM) was used as the anolyte, with its detailed composition provided in the Supplementary Information. The cathodic chamber contained 0.1 M potassium hexacyanoferrate (III) prepared in 0.1 M sodium phosphate buffer with the initial pH of both chambers adjusted to 7.0.

### MFC setup

H-type glass chambers with a total volume of 250 mL and a working volume of 200 mL were used. The anodic and cathodic chambers were separated by a CMI 7000 cation-exchange membrane (Membranes International, New Jersey, USA), tightly sealed with rubber gaskets and metallic clips to prevent leakage. Both chambers were equipped with carbon fibre electrodes measuring 5 × 5 cm (25 cm²) connected through a 2.2 kΩ resistor (R_ext_), which served as the fixed operational load for continuous voltage monitoring. External resistance is an important operational parameter in MFCs as it regulates electron flow through the external circuit and consequently influences cell voltage, current generation, biofilm behaviour and overall bioelectrochemical performance (López Zavala and Cámara Gutiérrez 2023; Xu *et al*. 2024; Potrykus *et al*. 2025). Experimental studies have demonstrated that MFC performance depends on the relationship between external resistance (R_ext_) and internal resistance (R_int_), with power output generally favoured when the external load approaches the internal resistance of the system (López Zavala and Cámara Gutiérrez 2023; Xu *et al*. 2024). The external resistance of 2.2 kΩ was selected based on the experimentally determined polarisation/resistance characteristics of the H-type MFC design used in this study. The characterisation of the internal resistance was done previously by Fernando *et al*. (2014) who showed that the maximum power density was obtained at an external resistance of 2.2 kΩ. For electrochemical characterisation, R_ext_ was subsequently decreased from 1 MΩ to 4 Ω during polarisation analysis to evaluate the voltage-current response and determine power output. Variation of the external electrical load is an established approach for characterising MFC performance because the measured voltage, current and power output are dependent on the applied load (Potrykus *et al*. 2025). The system was maintained in a Stuart 16D incubator (Fisher Scientific, UK) set at 30 °C. Voltage-time data was continuously recorded using a PicoLog data acquisition software (Pico Technology, UK). Experiments were conducted in quadruplicate to ensure reproducibility and reliability.

### Experimental set up

60 mg of sterilised 4 × 4 mm cut amorphous PET (0.25 mm thick) pieces were used as the sole carbon source in the study. A 40-day experiment compared PET biodegradation in the control (abiotic - no microbes added), *Ideonella sakaiensis* monoculture, *Shewanella oneidensis* monoculture, and a coculture of *Ideonella sakaiensis* and *Shewanella oneidensis*. Samples collected after 40 days were analysed following washing with 1% (w/v) sodium dodecyl sulphate (SDS) to remove attached biomass and residual organic material, followed by thorough rinsing with water and overnight drying under aseptic conditions prior to analysis.

## Analytical methods

### PET microplastic characterisation

#### FTIR spectroscopy

Functional groups in neat PET, the control, and post-biodegradation samples were analysed using UATR-FTIR (Perkin-Elmer) at 90 force units over the range 4000–450 cm⁻¹, with spectra averaged from 3 scans.

#### SEM imaging

Surface morphology of microplastics post-treatment was examined via SEM (Jeol JSM-6480LV) after gold coating in a vacuum environment. By adjusting the aperture dials and the x- and y-stigmators, the device was set to produce a clear image.

#### Biofilm assay

Microbial colonisation on PET was quantified using crystal violet assay, in which samples were stained with 0.1% (w/v) Crystal Violet for 15 min, washed with 5% (v/v) acetic acid, and absorbance was measured at 595 nm.

### Biodegradation Evaluation in MFC

#### Gravimetric Weight Loss

Overnight dried PET microplastics obtained from post-biodegradation were weighed and recorded. PET weight loss (%) was quantified as:

$$\mathrm{W}\mathrm{L}\:\left({\%}\right)=\frac{{\mathrm{I}}_{0}-{\mathrm{I}}_{\mathrm{F}}}{{\mathrm{I}}_{0}}\:\mathrm{x}\:\:100$$

Where I_o_ is the initial PET weight, and I_F_ is the final PET weight post-biodegradation.

As gravimetric measurements may be influenced by residual surface-associated material, mass-loss values were interpreted alongside complementary analytical measurements.

#### Soluble COD

The production of soluble organics in solution was measured using the Hach COD TNT, 20–1500 mg/L O2 vial (UK). 2 mL of supernatant was digested at 150 °C for 2 h, cooled, and analysed at 605 nm using a Lange DR3900 spectrophotometer. The reduction of dichromate to Cr^3+^ were quantified spectrophotometrically, and converted to sCOD using the equation:

$$\:\mathrm{s}\mathrm{C}\mathrm{O}\mathrm{D}\:\left(\mathrm{m}\mathrm{g}/\mathrm{L}\:{\mathrm{O}}_{2}\:,\:\mathrm{d}\mathrm{a}\mathrm{y}\:40\right)=\boldsymbol{C}\boldsymbol{O}{\boldsymbol{D}}_{\boldsymbol{s}}\:\left(\mathrm{a}\mathrm{f}\mathrm{t}\mathrm{e}\mathrm{r}\:\mathrm{b}\mathrm{i}\mathrm{o}\mathrm{d}\mathrm{e}\mathrm{g}\mathrm{r}\mathrm{a}\mathrm{d}\mathrm{a}\mathrm{t}\mathrm{i}\mathrm{o}\mathrm{n}\right)\:-\:\boldsymbol{C}{\boldsymbol{O}\boldsymbol{D}}_{0}\left(\mathrm{m}\mathrm{e}\mathrm{d}\mathrm{i}\mathrm{a}\right)$$

#### GC-FID

Supernatants from days 10, 20, 30, and 40 were analysed for metabolites using a Thermofischer Scientific Trace 1600 gas chromatograph (GC) coupled with a flame ionisation detector (FID) and an automated liquid sampler with a 30 (5 min) – 170 °C (15 min) oven ramp and a 250 °C detector. GC temperature was set to 220 °C with a constant helium flow rate of 3 mL min⁻¹.

### Electrochemical performance analysis

#### Voltage and current output over time

Voltage (V) across the MFC was continuously recorded using a PicoLog data-acquisition system (Pico Technology, UK). Current (I) was calculated using Ohm’s law, I = V/R_ext_, where R_ext_ represents the external resistance. Open-circuit voltage (OCV) was measured without an external load to determine the maximum cell voltage under open-circuit conditions.

#### Power density curves

Power density was calculated from the measured voltage and current (P = VI) and normalised to the electrode surface area (0.0025 m²). Current was calculated using Ohm’s law (I = V/R_ext_). Power density (mW m⁻²) was plotted against current density (mA m⁻²) to determine the maximum power output of each MFC.

#### Polarisation curves

Polarisation analysis was performed using a resistance decade box (Tenma, Taiwan) after stable voltage was achieved across the 2.2 kΩ operational load. Following open-circuit voltage (OCV) measurement, external resistance was sequentially decreased from 1 MΩ to 4 Ω, and the corresponding voltage was recorded at each resistance. Current density was calculated by normalising the current to the electrode surface area, and voltage (mV) was plotted against current density (mA m⁻²) to construct the polarisation curves.

### Statistical analysis

Data (mean ± SD, *n* = 4) were analysed via ANOVA; Statistical significance was set at *p* < 0.05, with significance levels ranging from * to **** (*p* < 0.0001).

## Results

### PET microplastic characterisation

#### FTIR spectroscopy

**Fig. 2:**
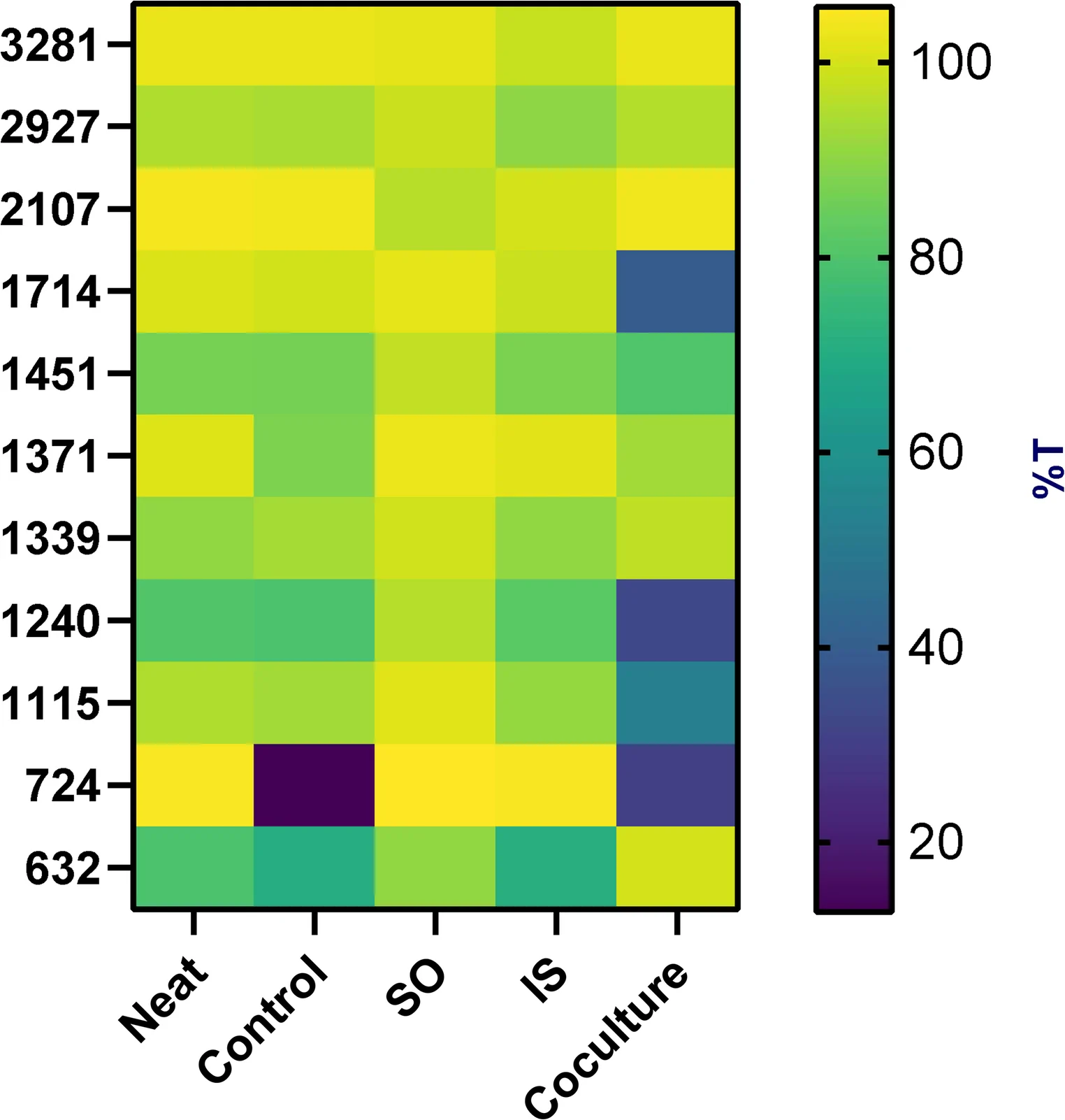
Heat map of FTIR analysis %transmittance over **a**) Neat PET, **b**) Control: PET with no organism, **c**) SO: *S. oneidensis* monoculture, **d**) IS: *I. sakaiensis* monoculture, **e**) Coculture: *I. sakaiensis* & *S. oneidensis*, *n* = 4

FTIR analysis revealed treatment-dependent physicochemical changes in PET following 40 days of MFC incubation (Fig. 2). Neat PET exhibited the characteristic spectral profile of PET, including bands associated with the ester carbonyl (~ 1722 cm⁻¹), ester-associated C–O/C–O–C vibrations (~ 1252–1101 cm⁻¹), and aromatic-associated fingerprint regions. Comparison with the abiotic control showed preservation of the principal PET spectral profile, with only minor changes in peak position and transmittance. The carbonyl band shifted slightly from ~ 1722 cm⁻¹ in neat PET to ~ 1721 cm⁻¹ in the abiotic control, while the ~ 1252 cm⁻¹ band shifted to ~ 1249 cm⁻¹. Other characteristic regions, including ~ 2912, 1426, 1329, 1101, 966 and 698 cm⁻¹, remained largely unchanged, indicating relatively limited physicochemical modification under abiotic conditions. This is consistent with the comparatively low PET mass loss (2.57%) observed in the abiotic control.

In contrast, the microbial treatments exhibited more distinct spectral alterations. The *I. sakaiensis* monoculture showed features at approximately 3282, 2924, 2854, 2107, 1725, 1648, 1426, 1329, 1248, 1100, 966, 698 and 612 cm⁻¹. Changes in the carbonyl (~ 1725 cm⁻¹) and ester-associated C-O regions (~ 1248 and 1100 cm⁻¹), together with the appearance/change around ~ 3282 cm⁻¹, indicate modification of carbonyl-, ester- and hydroxyl-associated surface functionalities. Such changes are consistent with microbial PET depolymerisation, which involves hydrolytic modification of ester bonds within the PET backbone (Liu *et al*. 2025). Notably, PET exposed to *S. oneidensis* MR-1 also exhibited a distinct spectral profile, with prominent features at approximately 2923, 2105, 1426, 1329, 1248, 1101, 965, 698 and 608 cm⁻¹. Alterations within the ~ 1248 and ~ 1101 cm⁻¹ ester-associated regions provide complementary evidence of PET surface modification. A feature around 2105–2107 cm⁻¹ was observed in both monocultures, representing an additional treatment-associated spectral alteration. As PET modification by wild type *S. oneidensis* MR-1 remains largely unexplored, these findings provide additional evidence of its interaction with PET under the conditions investigated, although the underlying biochemical mechanism remains to be established. The coculture displayed pronounced absorption features at approximately 1714, 1240, 1116, 1094 and 725 cm⁻¹. Changes in the ester carbonyl (~ 1714 cm⁻¹) and C–O/C–O–C-associated regions (~ 1240–1094 cm⁻¹) indicate substantial modification of ester-containing functionalities, while the prominent ~ 725 cm⁻¹ feature indicates alteration within the aromatic-associated structural environment of PET. Interestingly, the ~ 2105–2107 cm⁻¹ feature observed in the individual monocultures was not prominent in the coculture, demonstrating treatment-dependent differences in PET surface chemistry. Overall, the greater spectral alterations in microbial treatments relative to the abiotic control support microbial-associated physicochemical modification of PET. Individual FTIR spectra for neat PET, abiotic control, *S. oneidensis* monoculture, *I. sakaiensis* monoculture and coculture, together with the combined overlaid spectra, are provided in the Supplementary Information (Fig. S1 – S6).

#### SEM analysis

**Fig. 3:**
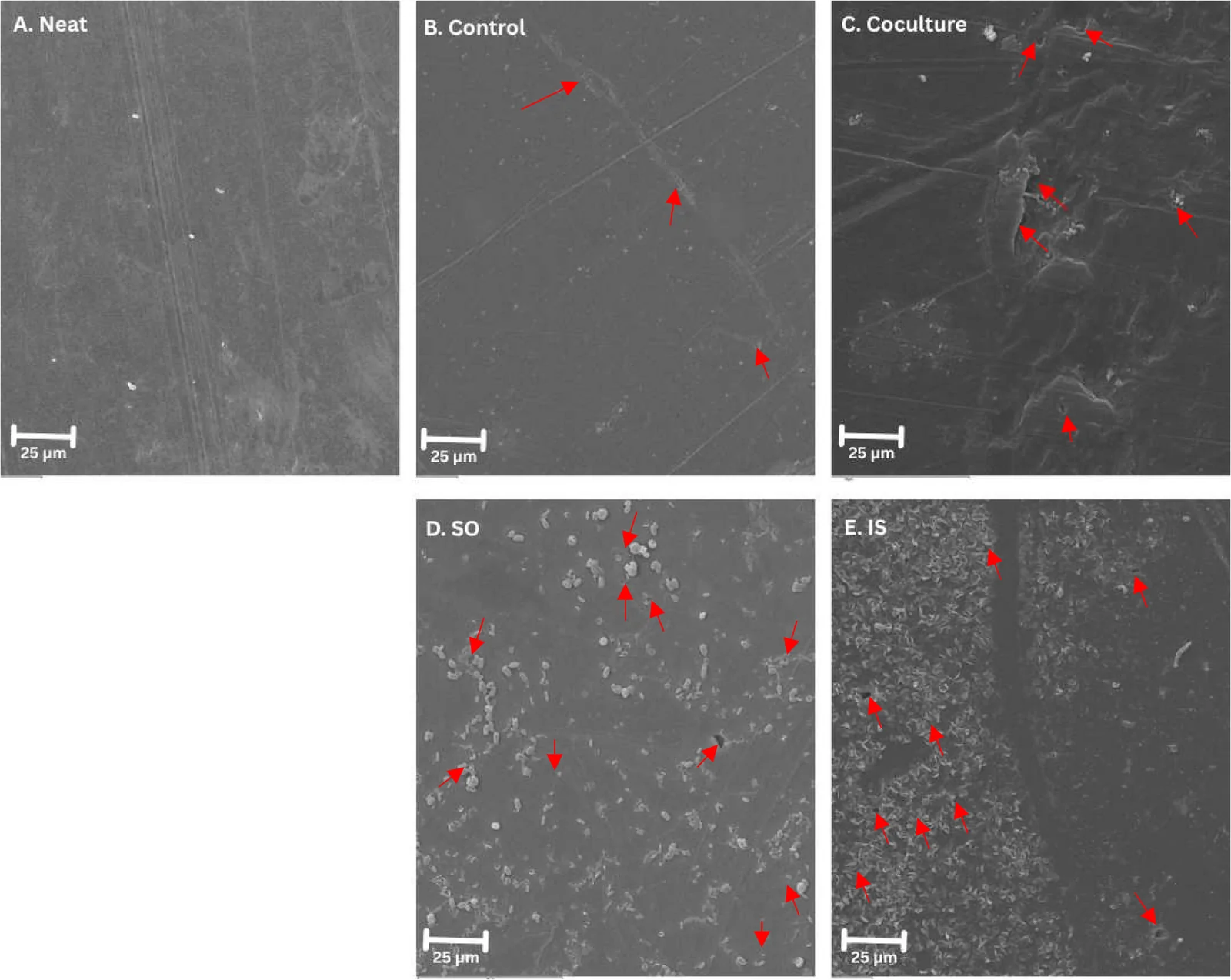
SEM micrographs (×1200 magnification; scale bar = 25 μm) showing PET surface morphology after 40 days of MFC incubation: (**A**) neat PET, (**B**) abiotic control, (**C**) *I. sakaiensis - S. oneidensis* coculture, (**D**) S. *oneidensis* monoculture, and (**E**) *I. sakaiensis* monoculture. Red arrows indicate representative areas of surface alteration

SEM revealed treatment-dependent morphological changes in PET following 40 days of MFC incubation (Fig. 3). Neat PET exhibited a relatively smooth and uniform surface (Fig. 3A), whereas the abiotic control showed only minor surface irregularities and shallow abrasions (Fig. 3B), indicating limited abiotic alteration. In contrast, coculture-treated PET displayed pronounced surface deterioration, including pits, crevices, fissures and layered erosion patterns, with comparatively little surface-associated microbial debris evident in the analysed field (Fig. 3C). PET exposed to *S. oneidensis* MR-1 exhibited localised pits and cracks together with substantial surface-associated biofilm biomass and cellular debris that remained attached after 1% (w/v) SDS washing (Fig. 3D). *I. sakaiensis*-treated PET similarly showed pronounced surface alteration, including pores, pits and cracks (Fig. 3E). EPS-like material was evident across the microbial treatments, supporting microbial attachment and biofilm development on the PET surface. Persistent cellular debris was particularly prominent in the *S. oneidensis* treatment and comparatively less evident in the coculture and *I. sakaiensis* samples.

The persistence of surface-associated material also highlights an inherent limitation of gravimetric analysis, as residual EPS, biomass, cellular debris and/or inorganic deposits may contribute to the recovered PET mass and potentially underestimate the actual polymer mass loss (Afify *et al*. 2025). Overall, the greater morphological alterations observed in the microbial treatments relative to neat PET and the abiotic control, when considered alongside the gravimetric, FTIR and biofilm findings, support microbial-associated PET modification. The findings are consistent with PET biodegradation by *I. sakaiensis* and the coculture, while providing complementary evidence of PET-associated surface modification by *S. oneidensis* MR-1, for which the underlying mechanism remains to be established.

#### Biofilm assay

**Fig. 4:**
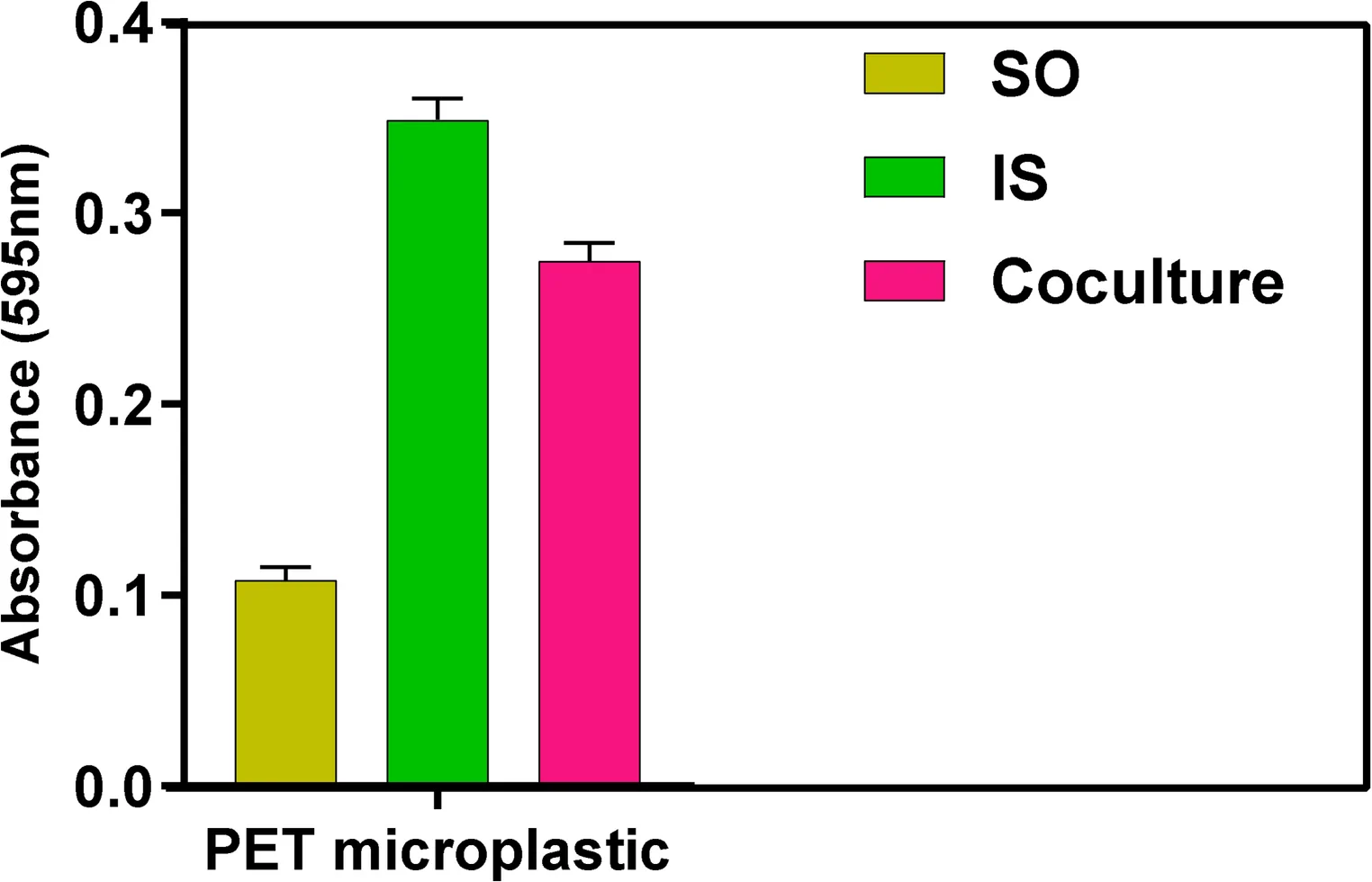
Biofilm formation on PET microplastics (surface area = 0.16 cm²) after 40 days, determined by crystal violet staining and expressed as absorbance at 595 nm (A₅₉₅; mean ± SD, *n* = 4). SO, *S. oneidensis* monoculture; IS, *I. sakaiensis* monoculture; Coculture, *I. sakaiensis* and *S. oneidensis*

Biofilm formation on PET varied across the microbial treatments (Fig. 4), with crystal violet absorbance (A₅₉₅) values of 0.10, 0.35 and 0.27 for *S. oneidensis*, *I. sakaiensis* and the coculture, respectively. The higher biofilm biomass observed for *I. sakaiensis* is consistent with its ability to colonise PET surfaces, potentially facilitating enzyme-substrate interactions during PET degradation (Song *et al*. 2024). The lower biofilm biomass in the coculture relative to the *I. sakaiensis* monoculture may reflect interspecies competition for PET surface attachment sites and differences in EPS production, potentially restricting uniform biofilm development. Similar behaviour has been reported in anaerobic bioelectrochemical systems, where *S. oneidensis* remained predominantly planktonic rather than becoming stably integrated within mature mixed-species biofilms (Engel *et al*. 2019). Overall, these findings demonstrate species- and community-dependent differences in PET-associated biofilm formation, which may contribute to the differences in PET modification observed among the treatments.

### Biodegradation evaluation in MFC

#### Gravimetric weight loss

**Fig. 5:**
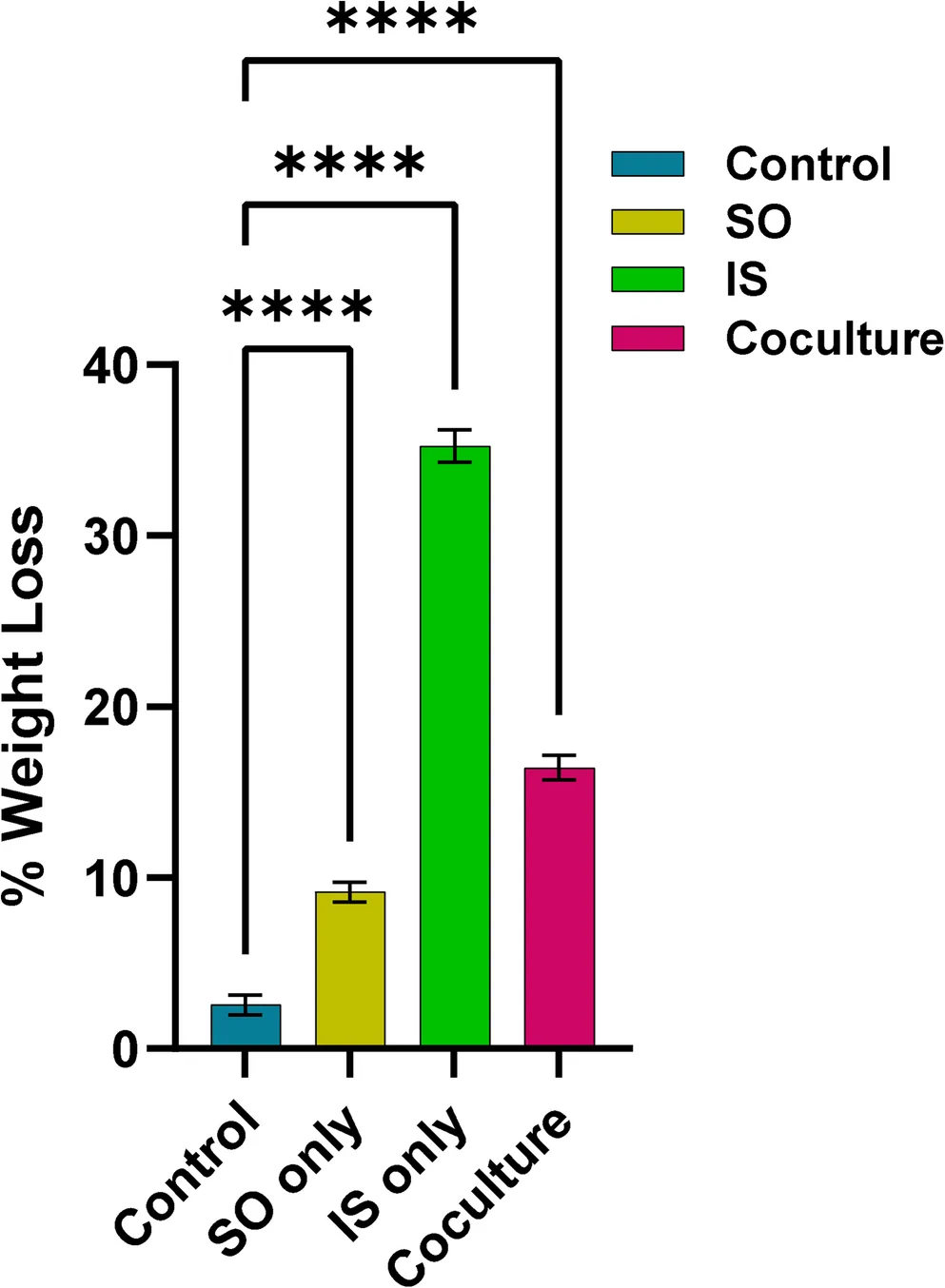
Indicates biodegradation weight loss percentage ± SD. Control: PET with no organism, SO: *S. oneidensis* monoculture, IS: *I. sakaiensis* monoculture, Coculture: *I. sakaiensis* & *S. oneidensis*, Statistical significance (Dunnett’s Multiple Comparison Test adjusted p-values) was determined using a one-way ANOVA: ns = not significant (*P* ≥ 0.05); *P  < 0.05; **P  < 0.01; ***P  < 0.001; ****P < 0.0001, *n* = 4)

After 40 days, the *I. sakaiensis* monoculture showed the highest PET mass loss (35.2%), followed by the coculture (16.4%) and *S. oneidensis* monoculture (9.16%), whereas the abiotic control showed substantially lower mass loss (2.57%) (Fig. 5). The limited loss in the abiotic control may reflect physicochemical weathering and leaching of non-covalently bound additives, oligomers and other low-molecular-weight components during prolonged incubation (Felsing *et al*. 2018; Bridson *et al*. 2023).

The substantially greater mass loss observed with *I. sakaiensis* is consistent with its established PETase–MHETase-mediated PET degradation pathway (Kalathil *et al*. 2022). Notably, *S. oneidensis* also showed greater mass loss than the abiotic control, indicating a PET-associated biological effect under the experimental conditions; however, the underlying mechanism remains to be established.

The lower PET mass loss in the coculture relative to the *I. sakaiensis* monoculture may reflect interspecies competition, metabolic imbalance and competition for PET surface colonisation, which can influence substrate utilisation and community performance (Iven *et al*. 2022; Engel *et al*. 2019; Song *et al*. 2024; McNichol *et al*. 2024). Future optimisation of microbial interactions, including spatial organisation, promotion of biofilm establishment and immobilisation of electroactive microorganisms, may enhance microbial colonisation, cooperative substrate utilisation and bioelectrochemical performance in coculture systems (Kalathil *et al*. 2022; Afify *et al*. 2025; Nyadroh *et al*. 2025).

#### Soluble COD

**Fig. 6:**
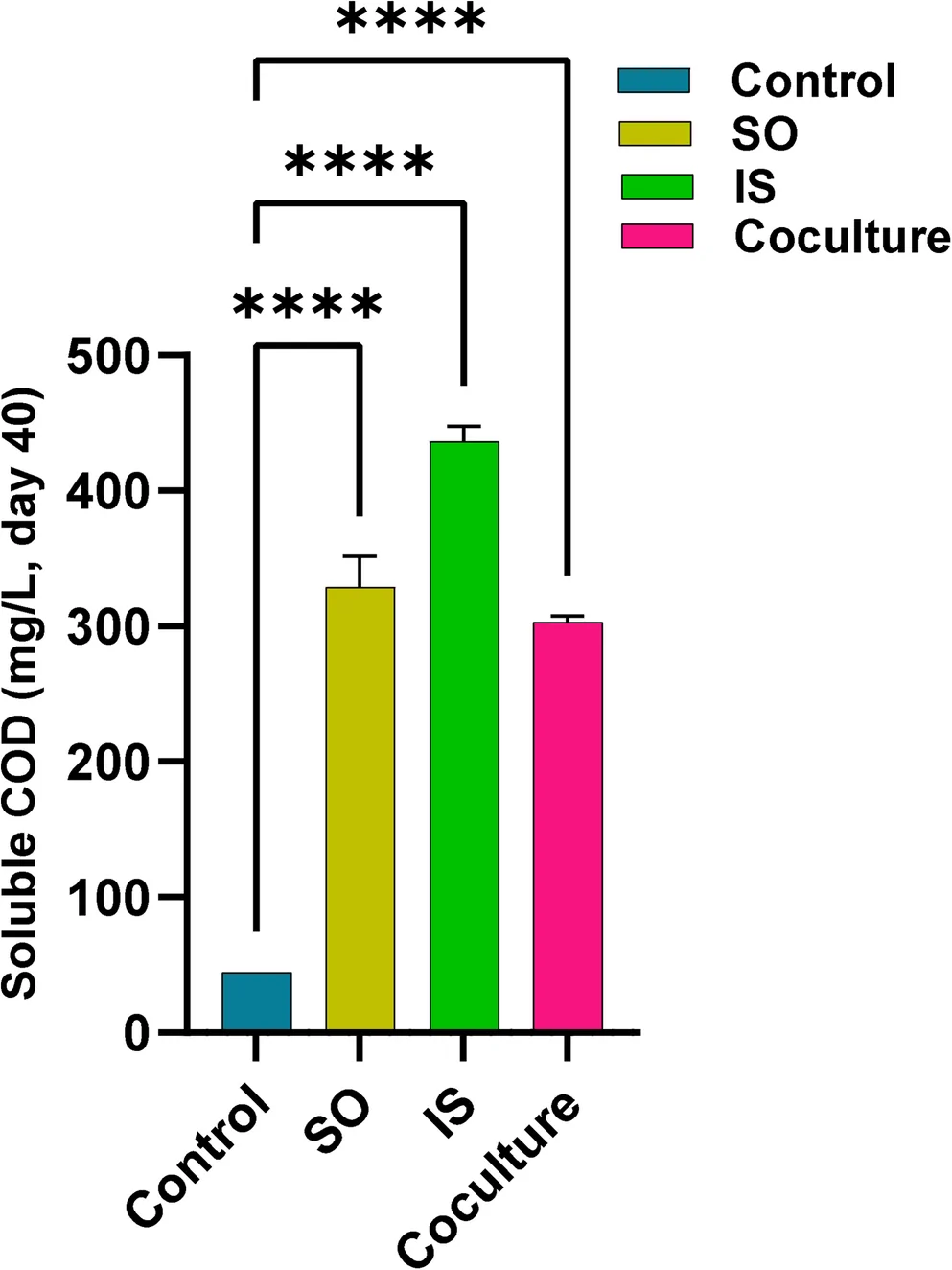
Soluble COD of study ± SD. Control: PET with no organism, SO: *S. oneidensis* monoculture, IS: *I. sakaiensis* monoculture, Coculture: *I. sakaiensis* & *S. oneidensis*, Statistical significance (Dunnett’s Multiple Comparison Test adjusted p-values) was determined using a one-way ANOVA: ns = not significant (*P*  ≥ 0.05); *P  < 0.05; **P  < 0.01; ***P  < 0.001; ****P  < 0.0001, *n* = 4)

Soluble chemical oxygen demand (sCOD), an indicator of soluble biodegradation intermediates, was lowest in the abiotic control (44.85 mg/L). All microbial treatments showed significantly higher sCOD values (Fig. 6), with the highest observed in the *I. sakaiensis* monoculture (433.36 mg/L), followed by *S. oneidensis* (328.8 mg/L) and the coculture (303.15 mg/L). Elevated sCOD in monocultures indicates greater accumulation of soluble PET degradation products under carbon-limited conditions. Integration of electroactive microbes into electrode-based systems may further enhance metabolite conversion and energy recovery by coupling microbial metabolism with electrochemical processes (Fang *et al*. 2020).

#### GC-FID

**Fig. 7:**
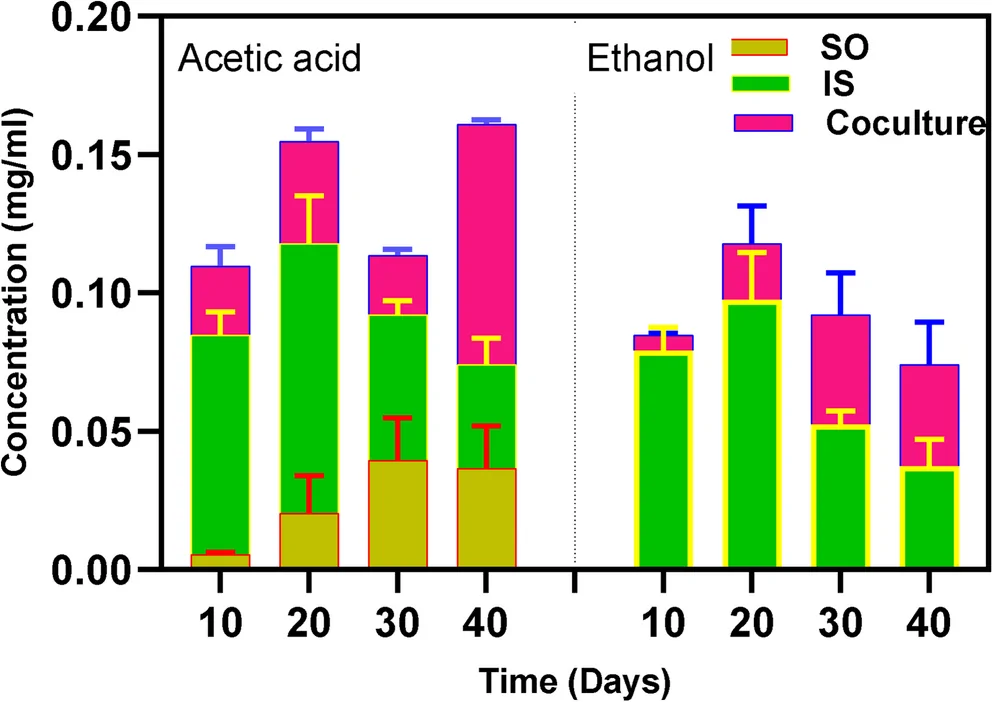
Acetic acid and ethanol concentration (mg/ml) ± SD. SO: *S. oneidensis* monoculture, IS: *I. sakaiensis* monoculture, Coculture: *I. sakaiensis* & *S.oneidensis*, *n* = 4)

GC-FID analysis detected acetic acid in the *S. oneidensis* monoculture, *I. sakaiensis* monoculture and coculture, whereas ethanol was detected only in the *I. sakaiensis* monoculture and coculture (Fig. 7). Neither metabolite was detected in the abiotic control. Representative GC-FID chromatograms, together with the corresponding acetic acid and ethanol standards used for retention-time comparison, are provided in the Supplementary Information (Figs. S7–S11).

Acetic acid concentrations reached maxima of 0.04 mg mL⁻¹ on day 30 in the *S. oneidensis* monoculture and 0.12 mg mL⁻¹ on day 20 in the *I. sakaiensis* monoculture, whereas the coculture showed progressive accumulation to 0.20 mg mL⁻¹ on day 40. Ethanol in the *I. sakaiensis* monoculture peaked at 0.09 mg mL⁻¹ on day 20 before declining to 0.04 mg mL⁻¹ by day 40. In the coculture, ethanol increased more gradually to 0.09 mg mL⁻¹ on day 30, followed by a slight decline to 0.07 mg mL⁻¹ on day 40. These treatment-dependent profiles indicate differences in metabolite production and/or utilisation between the monoculture and coculture systems.

### Electrochemical performance analysis

#### Voltage and current output over time

**Fig. 8:**
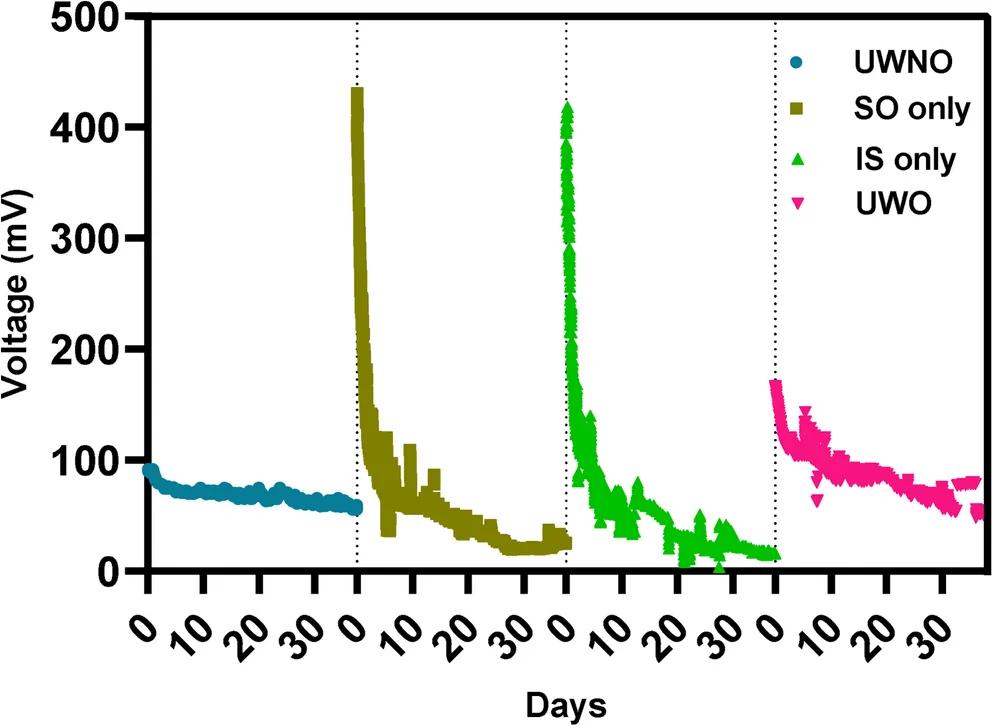
Average voltage output (mV) over 40 days of PET-fed MFC operation. Control: abiotic PET without microbial inoculation; SO: *S. oneidensis* monoculture; IS: *I. sakaiensis* monoculture; Coculture: *I. sakaiensis - S. oneidensis*. Data are presented as mean ± SD (*n* = 4)

Voltage output varied across the MFC treatments during the 40-day incubation, reflecting differences in bioelectrochemical activity over time (Fig. 8). The abiotic control showed a gradual decline from 91.5 to 56.5 mV. Higher initial voltages were observed in the *S. oneidensis* (430.33 mV) and *I. sakaiensis* (418 mV) monocultures, but these declined substantially to 28 and 16.5 mV, respectively, by day 40. In contrast, the *I. sakaiensis* - *S. oneidensis* coculture exhibited a comparatively more sustained voltage profile, decreasing from 167 to 50.5 mV over the experimental period. Overall, the treatment-dependent voltage profiles demonstrate measurable bioelectrochemical activity during PET incubation, although the underlying mechanisms responsible for electron transfer were not determined in the present study.

#### Power density curves

**Fig. 9:**
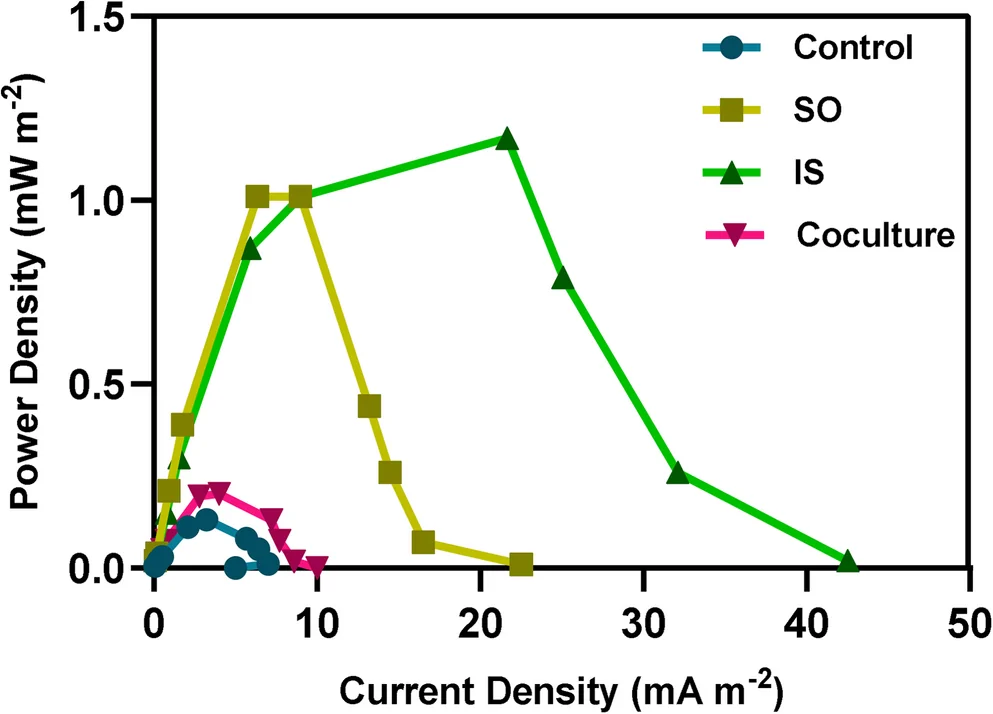
Power density - current density curves of PET-fed MFCs after 40 days of incubation. Control: abiotic PET without microbial inoculation; SO: *S. oneidensis* monoculture; IS: *I. sakaiensis* monoculture; Coculture: *I. sakaiensis - S. oneidensis*. Data are presented as mean ± SD (*n* = 4)

Power density varied markedly among the microbial treatments (Fig. 9). The *I. sakaiensis* monoculture achieved the highest maximum power density of 1.17 mW m⁻² at 21.64 mA m⁻², followed by the *S. oneidensis* monoculture at 1.01 mW m⁻² at 9 mA m⁻². In contrast, the coculture exhibited a substantially lower maximum power density of 0.202 mW m⁻² at 4 mA m⁻². The reduced coculture performance may reflect less favourable interspecies interactions, biofilm organisation and/or metabolic competition affecting bioelectrochemical activity. As the specific EET mechanisms were not investigated in this study, the observed differences in power output were not attributed to a particular electron-transfer pathway.

#### Polarisation curves

**Fig. 10:**
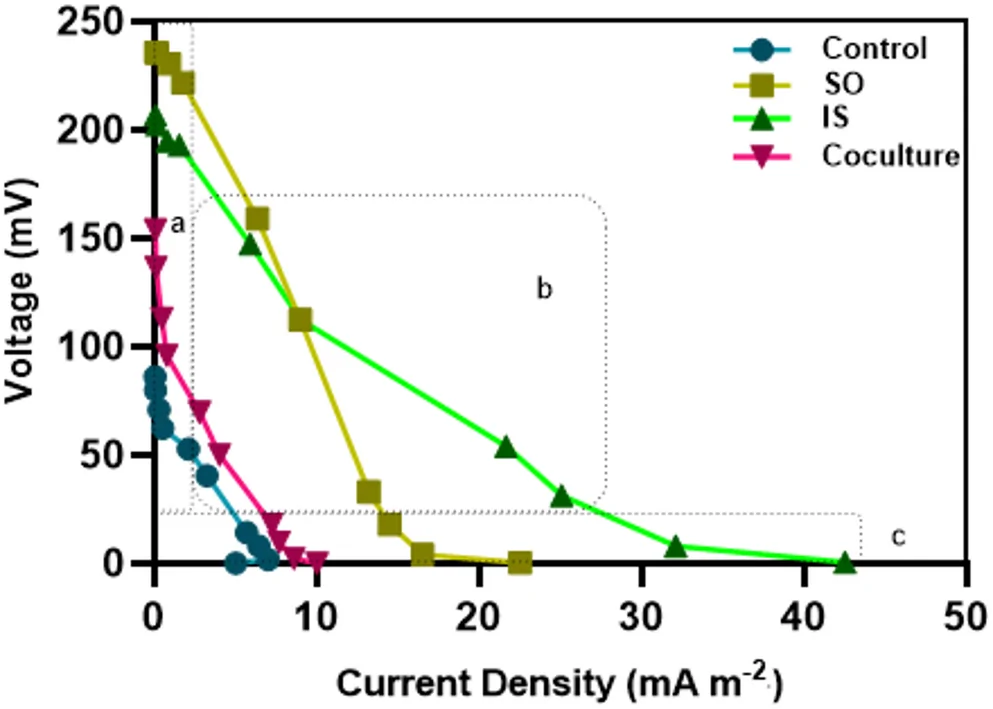
Polarisation curves showing voltage (mV) as a function of current density (mA m⁻²) for PET-fed MFCs. Regions (**a**), (**b**), and (**c**) represent the activation, ohmic region and mass-transport losses, respectively. Control: abiotic PET without microbial inoculation; SO: *S. oneidensis* monoculture; IS: *I. sakaiensis* monoculture; Coculture: *I. sakaiensis - S. oneidensis*. Data are presented as mean ± SD (*n* = 4)

Polarisation analysis showed distinct voltage - current density responses among the MFC treatments (Fig. 10). The abiotic control exhibited the lowest overall electrochemical performance, declining from 86.2 mV to 0.05 mV at 5 mA m⁻². The *S. oneidensis* monoculture decreased from 235.7 mV to 0.2 mV at 22.5 mA m⁻², while *I. sakaiensis* decreased from 206.9 mV to 0.4 mV at 42.5 mA m⁻². The coculture showed a lower response, decreasing from 153.9 mV to 0.1 mV at 1 mA m⁻². The observed profiles reflect treatment-dependent differences in MFC electrochemical performance. Although *S. oneidensis* has established EET capability, the specific electron-transfer mechanisms operating in these MFCs, including the electrochemical activity observed for *I. sakaiensis*, were not determined in the present study.

## Discussion

The present study demonstrates concurrent PET-associated biodegradation, metabolite formation and bioelectrochemical activity in MFCs operated with PET as the sole supplied carbon source. After 40 days, PET mass loss was highest for *I. sakaiensis* (35.2%), followed by the coculture (16.4%) and *S. oneidensis* (9.16%), compared with 2.57% in the abiotic control. Treatment-dependent FTIR changes, SEM-observed surface alterations and biofilm formation provided complementary evidence of microbial interaction with PET. However, because residual EPS, biomass and other surface-associated material may influence gravimetric measurements, these values are interpreted as PET mass loss rather than absolute polymer depolymerisation (Afify *et al*. 2025).

The comparatively high PET degradation by *I. sakaiensis* is consistent with its established PETase-MHETase-mediated hydrolytic activity (Yoshida *et al*. 2016) and its reported capacity for anaerobic PET fermentation to acetate and ethanol (Kalathil *et al*. 2022). Consistent with the latter, both metabolites were detected in the *I. sakaiensis* MFC. Notably, the monoculture also generated measurable electrochemical activity, reaching a maximum power density of 1.17 mW m⁻² at 21.64 mA m⁻². This differs from the system reported by Kalathil *et al*. (2022), in which *G. sulfurreducens* was employed as the exoelectrogen to utilise fermentation-derived acetate for electricity generation. As *I. sakaiensis* is not an established exoelectrogen, the electrochemical activity observed here represents an uncharacterised phenomenon requiring further mechanistic investigation.

Wild type *S. oneidensis* MR-1 also showed an unexpected PET-associated response, with 9.16% mass loss, acetate formation and a maximum power density of 1.01 mW m⁻² at 9 mA m⁻², accompanied by FTIR and SEM evidence of PET surface modification. Although *S. oneidensis* well characterised for extracellular electron transfer through cytochrome- and flavin-associated mechanisms (Zhang *et al*. 2023; Ford and TerAvest 2023), PET degradation by the wild type strain is not established. No PET-active enzymes were characterised in this study, nor was PET-derived carbon directly traced into acetate. Therefore, the findings are conservatively interpreted as evidence of PET-associated modification and acetate formation.

The coculture did not produce the anticipated synergistic response. Despite continued acetate and ethanol production, PET mass loss (16.4%) and maximum power density (0.202 mW m⁻² at 4 mA m⁻²) were lower than those of the best-performing monoculture. This may reflect competition for PET surface colonisation or nutrients, altered biofilm organisation, metabolic imbalance or inhibitory interspecies interactions (Engel *et al*. 2019; Iven *et al*. 2022; Song *et al*. 2024; McNichol *et al*. 2024).

Overall, the complementary gravimetric, FTIR, SEM, biofilm, sCOD, GC-FID and electrochemical analyses demonstrate distinct responses of *I. sakaiensis* and *S. oneidensis* to PET under MFC conditions. The principal contributions are the observation of electrochemical activity associated with *I. sakaiensis*, PET-associated modification and acetate formation by wild type *S. oneidensis*, and reduced PET degradation and power generation in their coculture. This study demonstrates the potential of MFCs as an integrated platform for PET microplastic bioremediation with simultaneous bioelectricity generation. Further isotope-tracing, enzymatic, and extracellular electron transfer (EET) studies are required to elucidate the mechanisms underlying PET transformation, metabolite formation, and electron transfer.

## Supplementary Information

Below is the link to the electronic supplementary material.

Supplementary Material 1

## Data Availability

No datasets were generated or analysed during the current study.

## Abbreviations

EET: Extracellular Electron Transfer
EPS: Extracellular Polymeric Substances
GC-FID: Gas Chromatograph With Flame Ionisation Detector
*I. sakaiensis*, IS: *Ideonella* *s* *akaiensis*
MFC: Microbial Fuel Cell
MNPs: Micro and Nano Particles
PET: Polyethylene Terephthalate
PETases: Polyethylene Terephthalate hydrolases
sCOD: Soluble Chemical Oxygen Demand
*S.** oneidensis*, SO: *She* *wanella* *o* *neidensis*
