# Supplementary material for: Biodegradation of Polyethylene Terephthalate (PET) microplastics in a Microbial Fuel Cell
Source: World J Microbiol Biotechnol. 2026 Nov 4;42(11):641. doi: 10.1007/s11274-026-05374-2 (PMC13736370; doi:10.1007/s11274-026-05374-2)
Supplement: Supplementary file 1 [file 11274_2026_5374_MOESM1_ESM.pdf]

Supplementary Material

Biodegradation of Polyethylene Terephthalate (PET) microplastics in a Microbial Fuel Cell

World Journal of Microbiology and Biotechnology

Merin Thyagu Pereira*, Sustainable Biotechnology Research Group, School of Life Sciences, University of Westminster, London, UK, m.thyagupereira@westminster.ac.uk, ORCID ID: 0009-0009-3126-2955

Tajalli Keshavarz, Sustainable Biotechnology Research Group, School of Life Sciences, University of Westminster, London, UK, T.Keshavarz@westminster.ac.uk

Godfrey Kyazze, Sustainable Biotechnology Research Group, School of Life Sciences, University of Westminster, London, UK, G.Kyazze@westminster.ac.uk

***Corresponding author:** Merin Thyagu Pereira

Table of Contents

1. Microorganisms and Culture Conditions…………………………………………………..2
2. Fourier Transform Infrared (FTIR) Spectroscopy………………………………………4
3. Gas Chromatography–Flame Ionisation Detection (GC–FID) Analysis…………………...6

1. Microorganisms and Culture Conditions:

Table 1: LB

Sl No:	Ingredients	Weight(g/L)
1	Tryptone	10
2	Yeast Extract	5
3	NaCl	10

Table 2: NBRC 802

Sl No:	Ingredients	Weight/L
1	Peptone	10 g
2	Yeast Extract	2 g
3	$MgSO_4.7H_2O$	1 g

Table 3: Bicarbonate Buffered Media (BBM)

Sl No:	Ingredients	Weight/L
1	NH_4Cl	1.50 g
2	Na_2PO_4	0.60 g
3	KCl	0.10 g
4	$NaHCO_3$	2.50 g
5	Trace element solution (see below)	10 ml
6	Vitamin solution (see below)	10 ml

Table 4: Sodium Phosphate Buffer, pH 7, 0.1M

Sl No:	Ingredients	Weight(g/L)
1	$NaH_2PO_4.H_2O$	5.14
2	Na_2HPO_4	8.91

Table 5: Trace Element Solution

Sl No:	Ingredients	Weight(mg/L)
1	Nitrilotriacetic acid	1500
2	$MnCl.4H_2O$	100
3	$FeSO_4.7H_2O$	300
4	$CoCl_2.6H_2O$	170
5	$ZnCl_2$	170
6	$CuSO_4.5H_2O$	40
7	$AlK(SO_4)_2.12H_2O$	5
8	H_3BO_4	5
9	$NaMoO_4$	90
10	$NiCl_2$	120
11	$NaWO_4.2H_2O$	20
12	$NaSeO_4$	100

Table 6: 100X Vitamin Mix Solution

Sl No:	Ingredients	Weight(mg/L)
1	P-aminobenzoic acid	50
2	L-Ascorbic acid	100
3	Folic acid	50
4	Riboflavin	10
5	Nicotinic Acid	100
6	Pantothenic Acid	100
7	Thiamine Hydrochloride	10
8	Biotin	100

Results:

2. Fourier Transform Infrared (FTIR) Spectroscopy

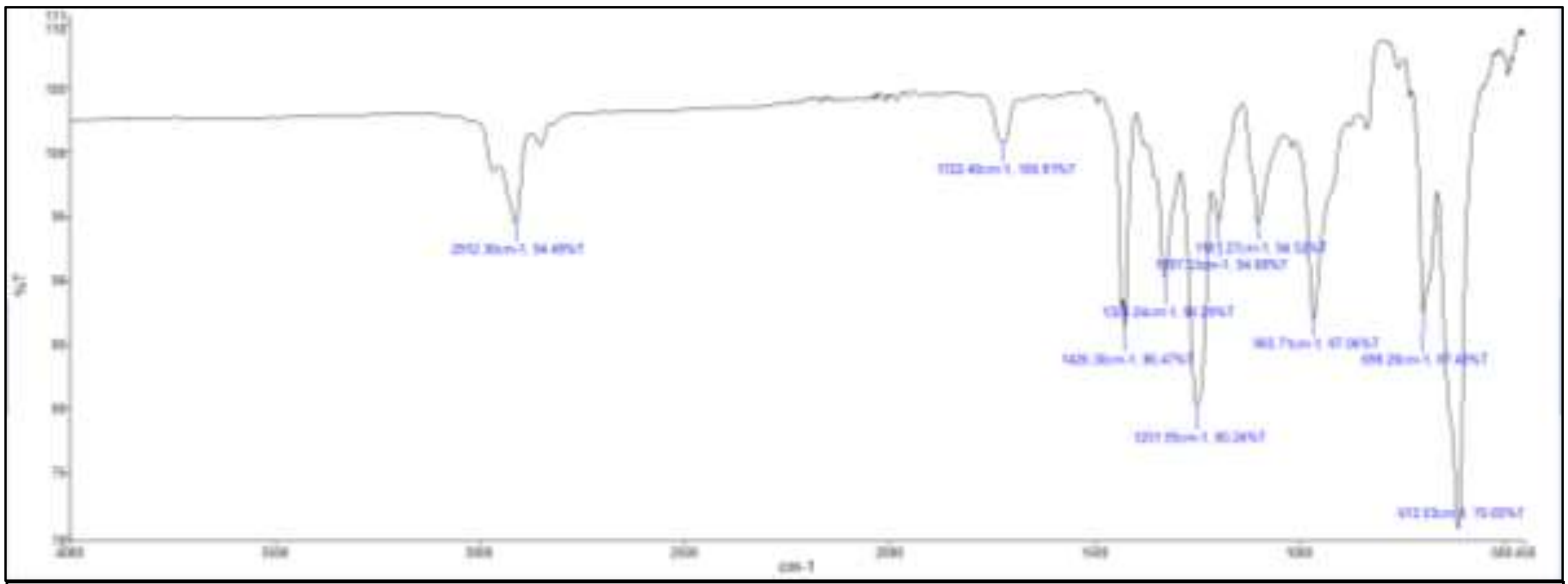

Fig. S1 - FTIR Spectrum of the Neat PET (black line).

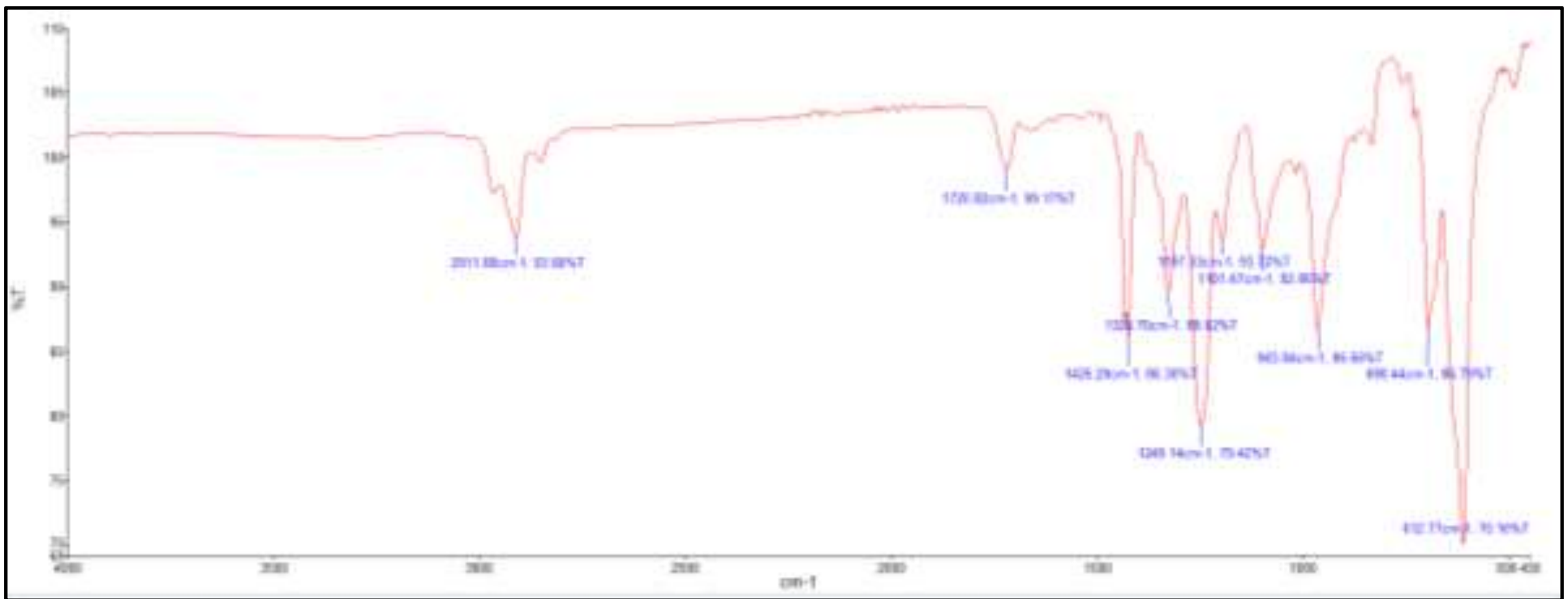

Fig. S2 - FTIR Spectrum of the Abiotic Control (Red line).

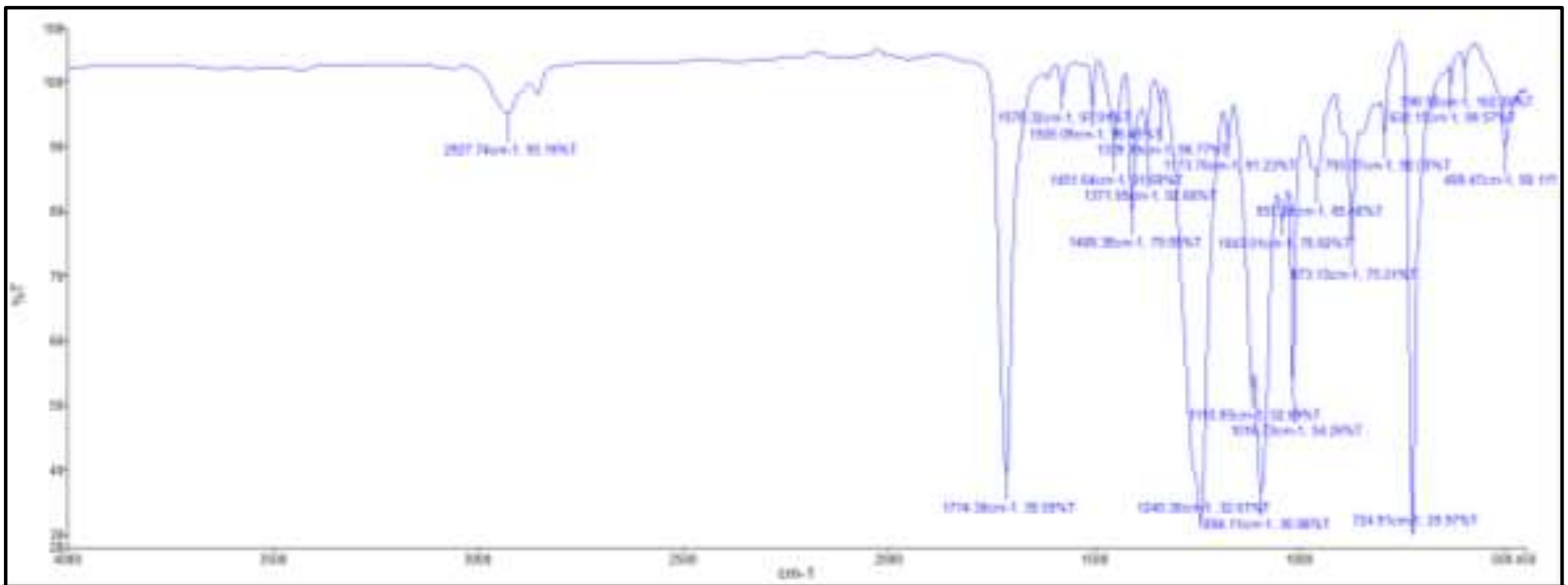

Fig. S3 - FTIR Spectrum of the Coculture (Blue line).

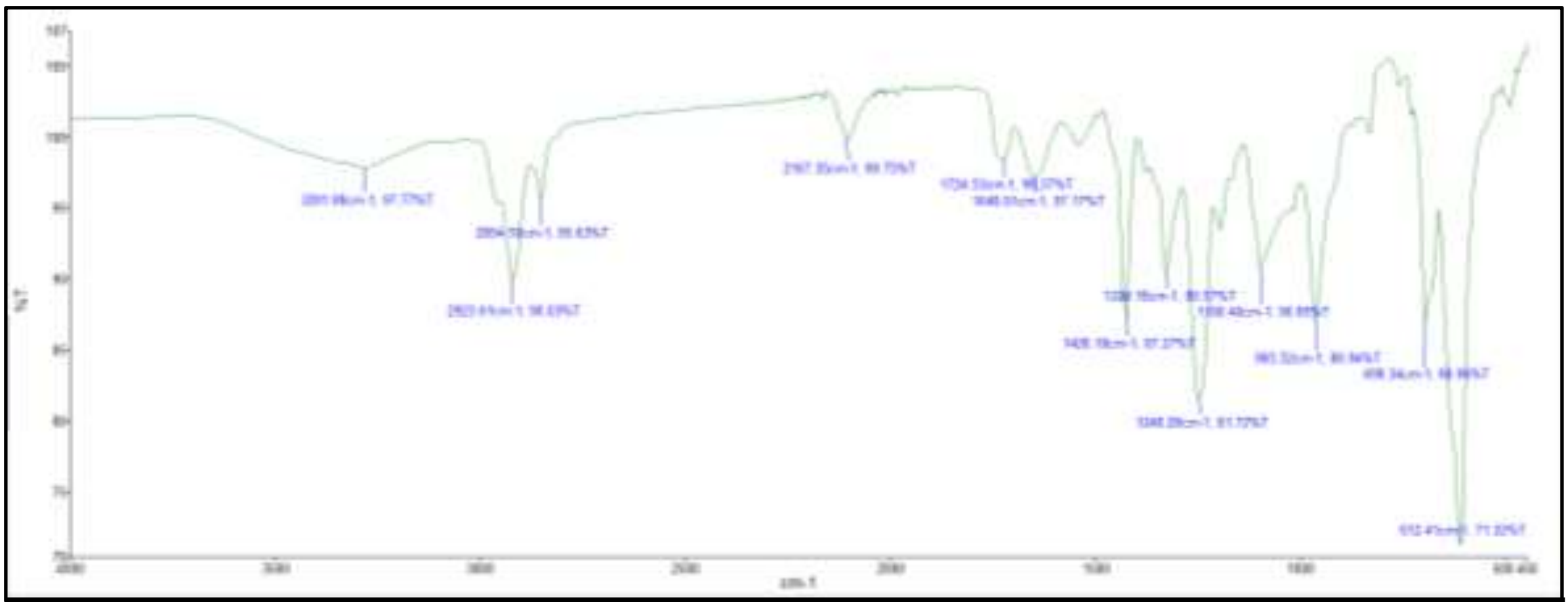

Fig. S4 - FTIR Spectrum of the *I. sakaiensis* monoculture (Green line).

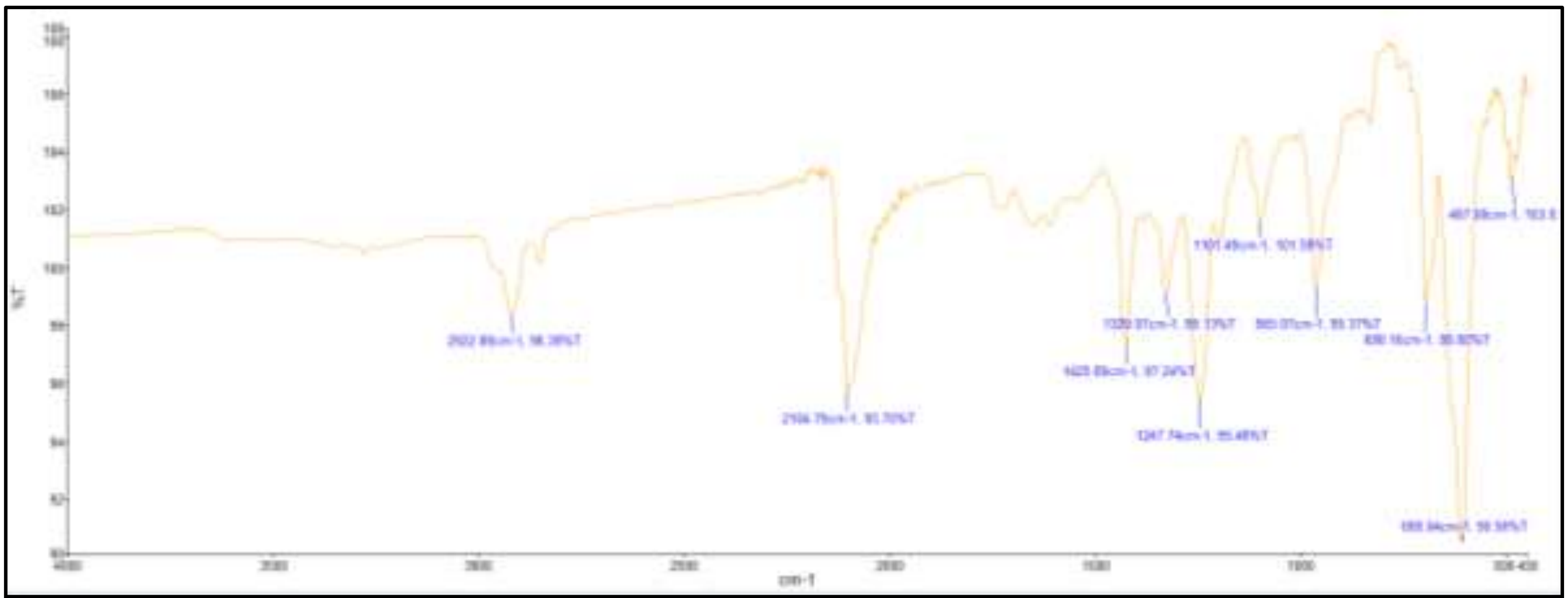

Fig. S5 - FTIR Spectrum of the *S. oneidensis* monoculture (Orange line).

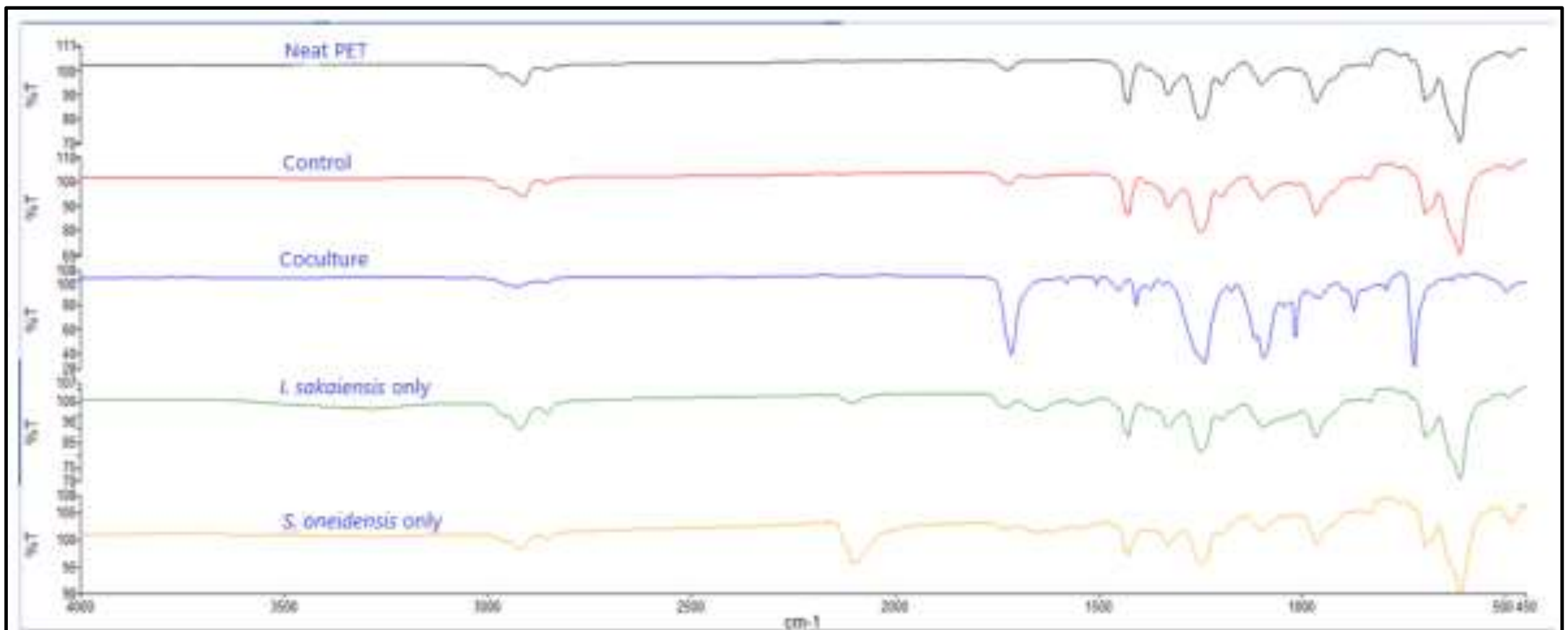

Fig. S6 - FTIR Spectrum of the Neat PET (black line), Abiotic Control (Red line), Coculture (Blue line), *I.sakaiensis* monoculture (Green line) and *S. oneidensis* (Orange line).

3. Gas Chromatography–Flame Ionisation Detection (GC–FID) Analysis:

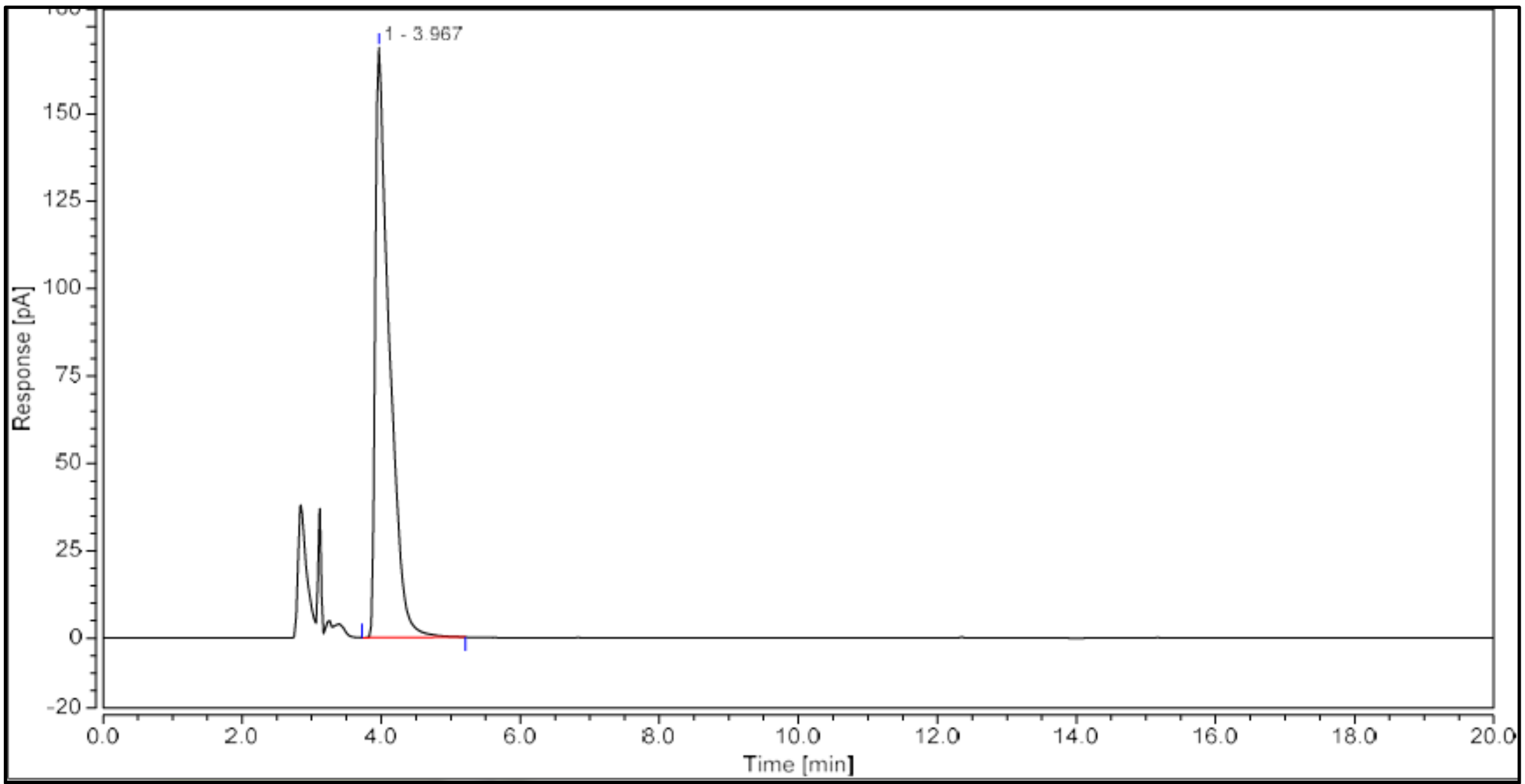

Fig. S7 - Representative GC-FID chromatogram of the ethanol standard, showing the characteristic ethanol peak at a retention time of approximately 3.9 min. This retention time was used as a reference for the identification of ethanol in experimental MFC samples analysed under the same chromatographic conditions.

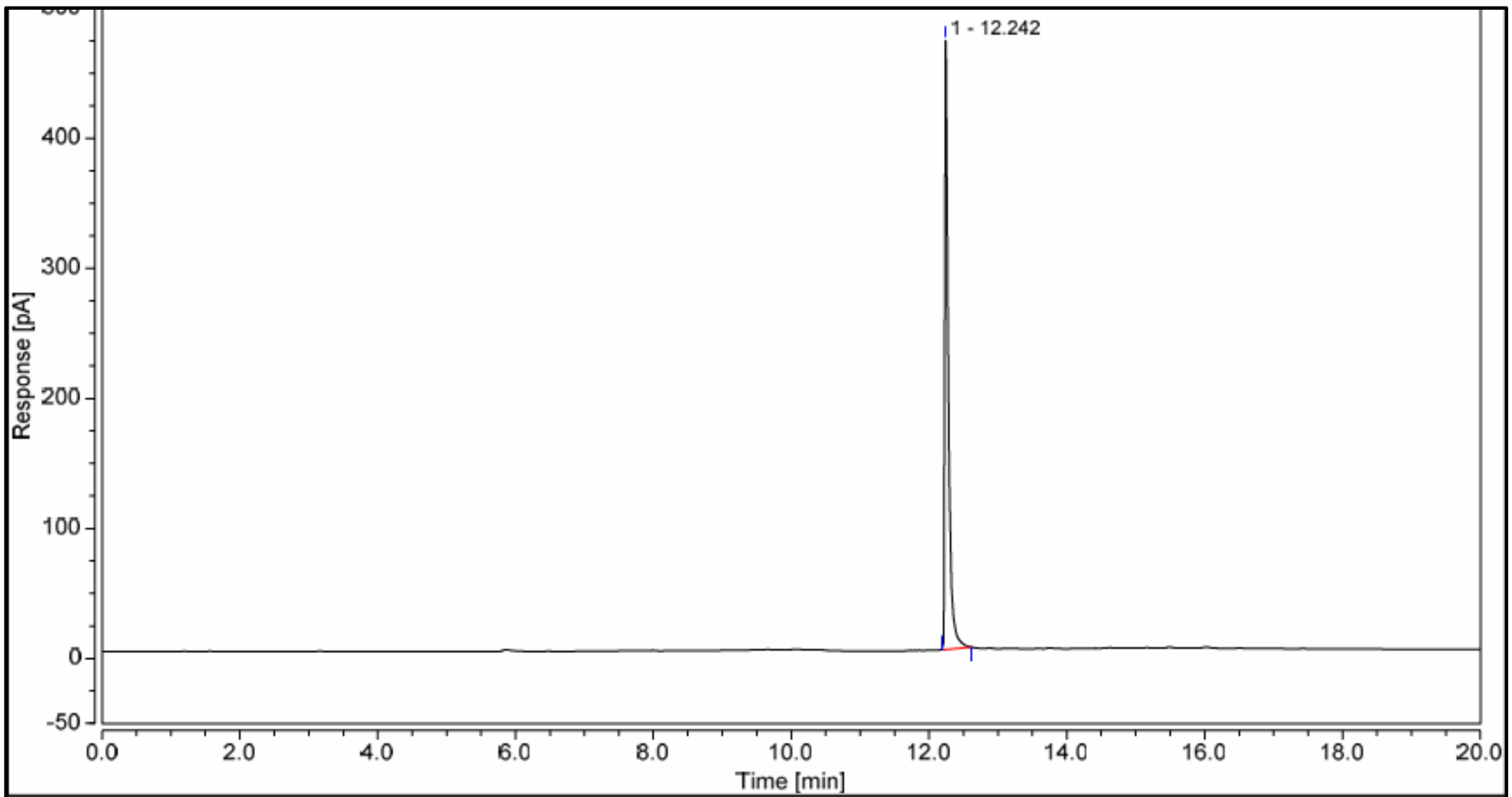

Fig. S8 - Representative GC-FID chromatogram of the acetic acid standard, showing the characteristic acetic acid peak at a retention time of approximately 12.24 min. This retention time was used as a reference for the identification of acetic acid in experimental MFC samples analysed under the same chromatographic conditions.

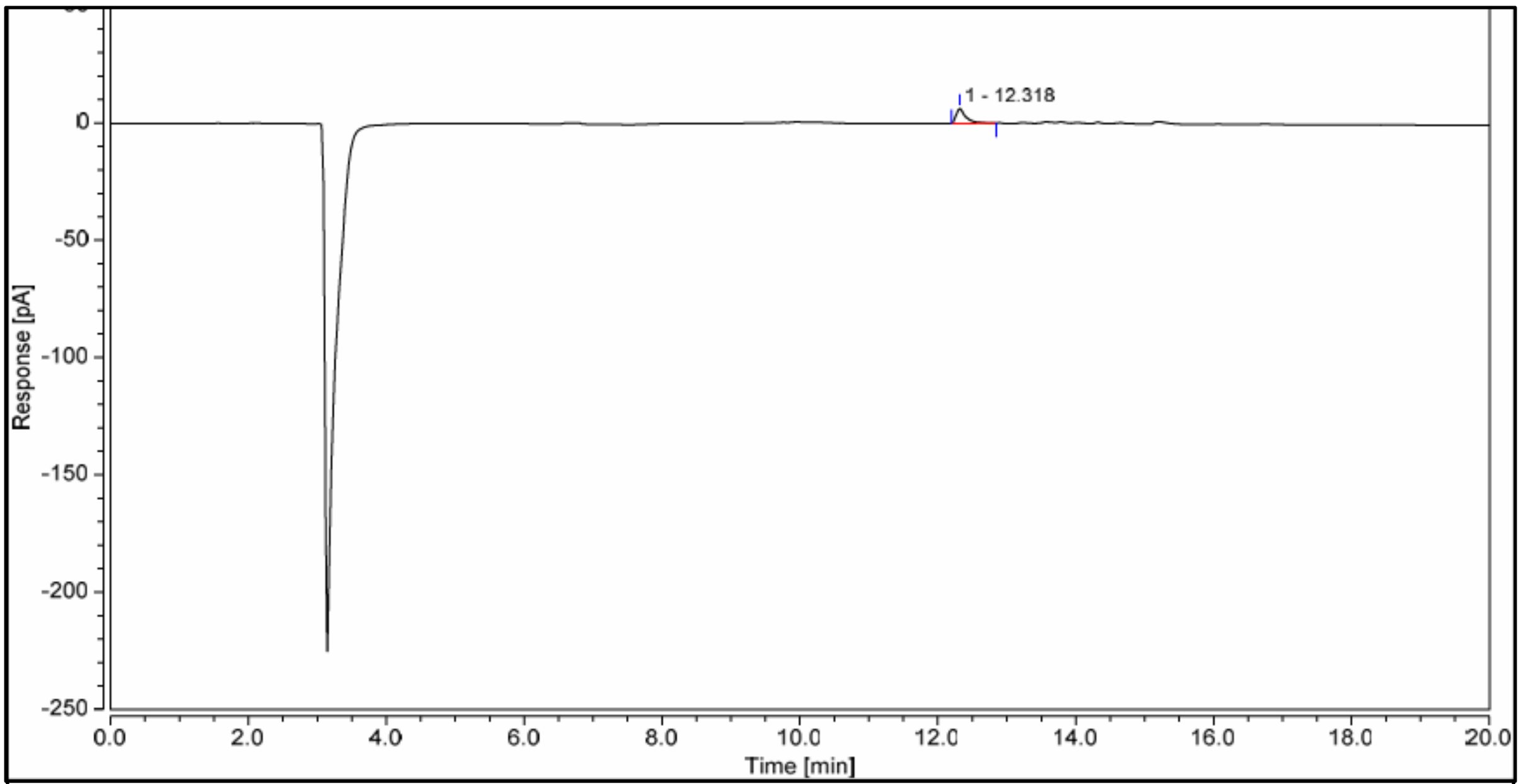

Fig. S9 - Representative GC-FID chromatogram of *S. oneidensis* MR-1. A peak was detected at a retention time of approximately 12.32 min, closely corresponding to the acetic acid standard (RT ≈ 12.24 min), supporting the identification of acetic acid in the experimental sample under the chromatographic conditions employed.

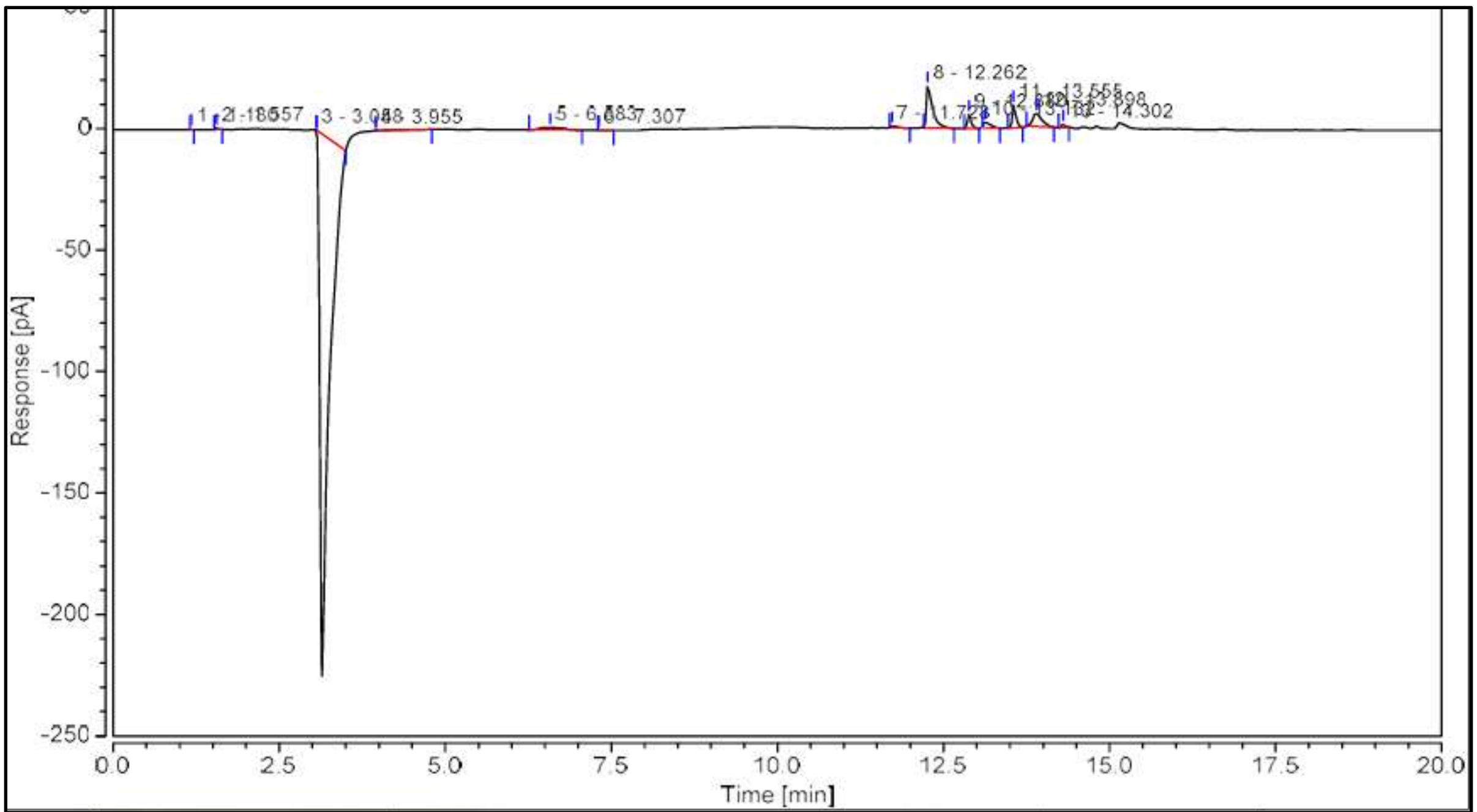

Fig. S10 - Representative GC-FID chromatogram of *I. sakaiensis*. Peaks were detected at retention times of approximately 3.96 min and 12.26 min, corresponding closely to the ethanol (RT ≈ 3.9 min) and acetic acid (RT ≈ 12.24 min) standards, respectively, supporting the identification of these metabolites in the experimental sample under the chromatographic conditions employed.

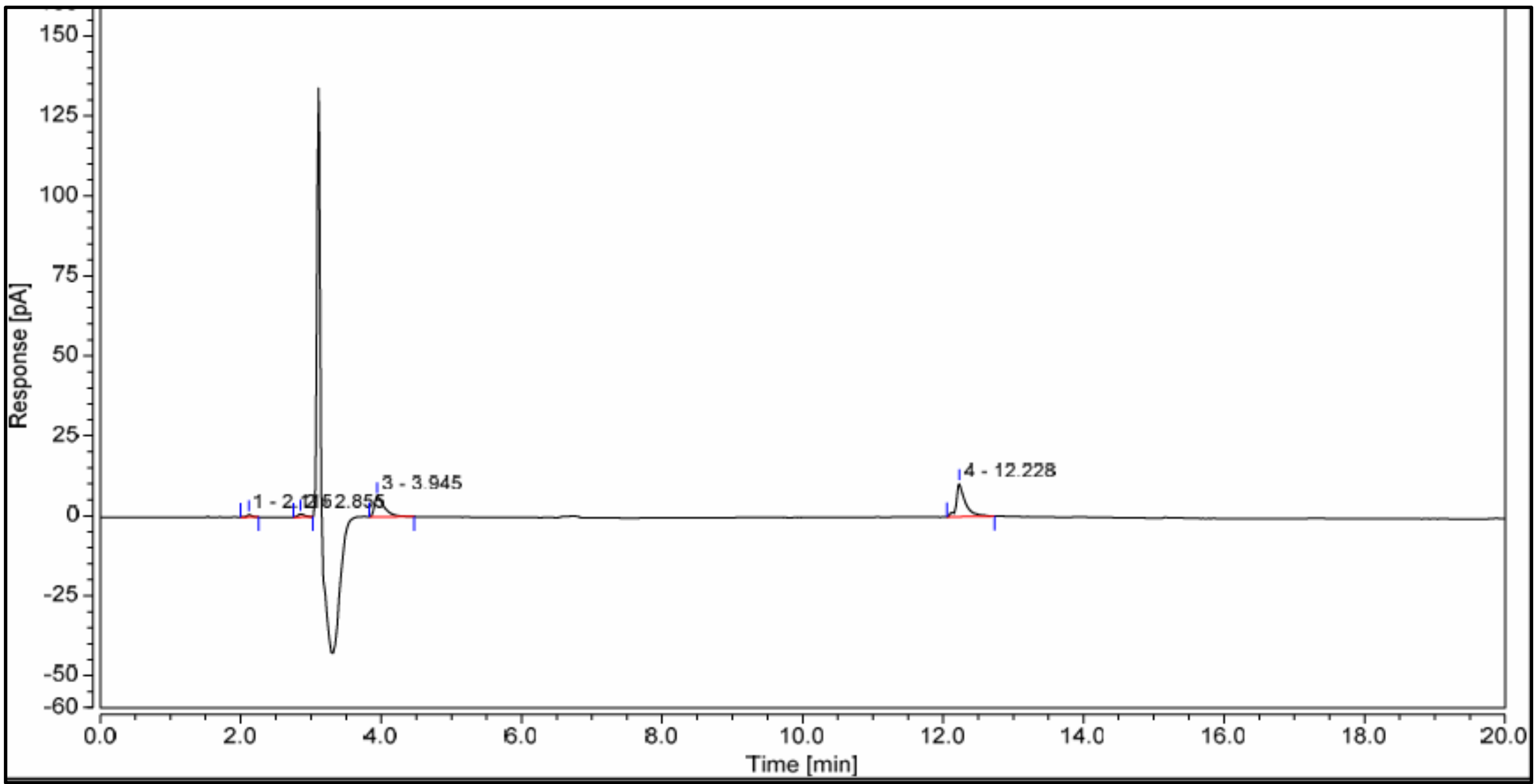

Fig. S11 - Representative GC-FID chromatogram of coculture. Peaks were detected at retention times of approximately 3.96 min and 12.22 min, corresponding closely to the ethanol (RT ≈ 3.9 min) and acetic acid (RT ≈ 12.24 min) standards, respectively, supporting the identification of these metabolites in the experimental sample under the chromatographic conditions employed.

Thank You!!
